# Higher-order patterns of aquatic species spread through the global shipping network

**DOI:** 10.1371/journal.pone.0220353

**Published:** 2020-07-31

**Authors:** Mandana Saebi, Jian Xu, Erin K. Grey, David M. Lodge, James J. Corbett, Nitesh Chawla

**Affiliations:** 1 Lucy Family Institute of Data and Society and Department of Computer Science and Engineering, University of Notre Dame, Notre Dame, Indiana, United States of America; 2 Citadel LLC, Chicago, Illinois, United States of America; 3 Division of Science, Mathematics, and Technology, Governors State University, University Park, Illinois, United States of America; 4 Cornell Atkins on Center for Sustainability and Department of Ecology and Evolutionary Biology, Cornell University, Ithaca, New York, United States of America; 5 College of Earth, Ocean and Environment, University of Delaware, Newark, Delaware, United States of America; 6 Lucy Family Institute of Data and Society and Department of Computer Science and Engineering, University of Notre Dame, Notre Dame, Indiana, United States of America; University of Pavia, ITALY

## Abstract

The introduction and establishment of nonindigenous species (NIS) through global ship movements poses a significant threat to marine ecosystems and economies. While ballast-vectored invasions have been partly addressed by some national policies and an international agreement regulating the concentrations of organisms in ballast water, biofouling-vectored invasions remain largely unaddressed. Development of additional efficient and cost-effective ship-borne NIS policies requires an accurate estimation of NIS spread risk from both ballast water and biofouling. We demonstrate that the first-order Markovian assumption limits accurate modeling of NIS spread risks through the global shipping network. In contrast, we show that higher-order patterns provide more accurate NIS spread risk estimates by revealing indirect pathways of NIS transfer using Species Flow Higher-Order Networks (SF-HON). Using the largest available datasets of non-indigenous species for Europe and the United States, we then compare SF-HON model predictions against those from networks that consider only first-order connections and those that consider all possible indirect connections without consideration of their significance. We show that not only SF-HONs yield more accurate NIS spread risk predictions, but there are important differences in NIS spread via the ballast and biofouling vectors. Our work provides information that policymakers can use to develop more efficient and targeted prevention strategies for ship-borne NIS spread management, especially as management of biofouling is of increasing concern.

## 1 Introduction

The subset of nonindigenous species (NIS) that become invasive pose significant and growing threats to ecosystems, infrastructure, human and animal health, resulting in an estimated annual cost of 1.4 trillion USD globally (of which 120 billion USD, 9% of global damages, is the estimated cost in the United States) [1]. In the Great Lakes alone, the annual cost of ship-borne NIS is estimated as high as 800 million USD [1]. While various mechanisms can lead to the introduction of NIS, this paper focuses on the NIS risks stemming from international commercial shipping, which is responsible for more marine NIS introduction around the world than any other mechanism [2–4], and whose risk rises with increasing trade and new shipping routes [5].

Prevention of NIS transfer and establishment has been identified as the most effective and economically efficient means to reduce NIS costs [6, 7]. However, cost-effective prevention requires a good understanding of the vectors of NIS introduction. Ship-borne NIS are transported via two main vectors: *ballast water* and *biofouling*. Ballast water management has received significant attention from researchers and policy-makers in recent years, including the International Maritime Organizations International Convention for the Control and Management of Ships’ Ballast Water and Sediments (BWM) and associated national legislation in the US. On the other hand, management of biofouling NIS transfer has received relatively less attention [8–10]. Not only do the two vectors of marine NIS transport require very different management interventions, but they also present challenges on how to effectively model their respective pathways to more accurately assess risk. Accurate risk assessment is critical to inform a cost-effective and efficient strategy for NIS risk management.

**Fig 1:**
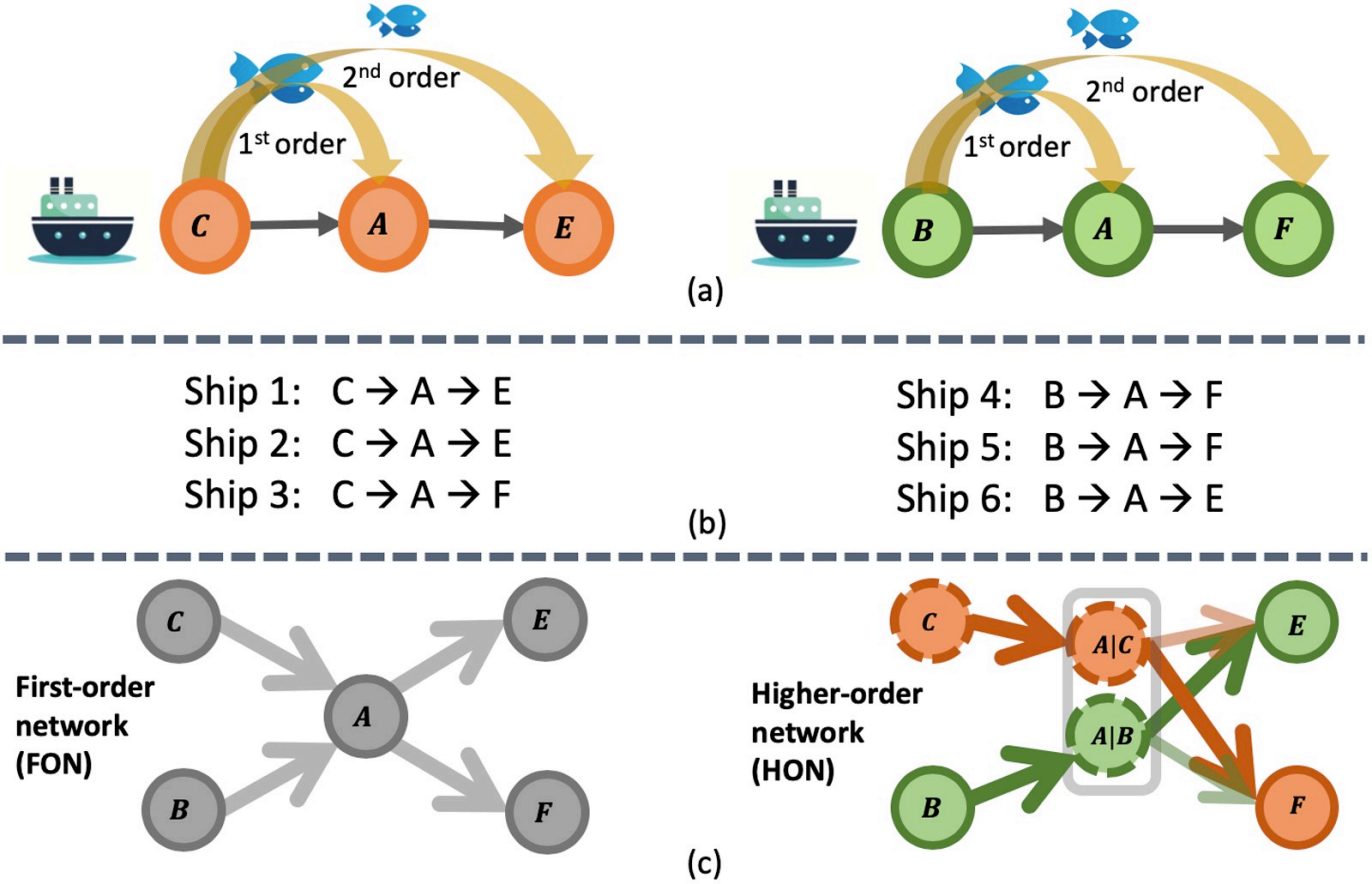
Two different patterns of higher-order species transfer between ports. (b) Out of six ships, ship 1 and 2 follow the pattern in (a) left, and ship 4 and 5 follow the pattern in (a) right. Conventional first-order network ((c) left), where organisms have the same probability of being introduced to port *E* and *F* via port *A*. Higher-order network ((c) right) with a second-order dependency example in species transfer. Due to higher-order patterns in ship movements, organisms native to port *C* are more likely to get transferred to port *F* via ships traveling through port *A* and organisms native to port *B* are more likely to get transferred to port *E* via ships traveling through port *A*.

We posit that NIS spread through the global shipping network is not a simple first-order Markov process as mostly assumed in the literature [11–15]. Rather, the pathways of NIS introduction exhibit **higher-order pathways of species transfer**, because many ships do not discharge their entire ballast water or release all of their biofouling species in their first port of call. For example, consider the case where a ship visits port *C*, *A*, and then *E*. Suppose the ship takes in ballast water in port *C*, and discharges a portion of water in port *A*, and discharges the rest in port *E*. This means that there is still a considerable risk of species introduction from port *C* to port *E*, in addition to port *A*. Fig 1(a) left displays an illustration of this situation. The first-order connections may have higher species transfer risk, but there is a non-trivial possibility of species transfer via either of the mentioned mechanisms from port *C* to port *A*, and *E*. Recurrence of such movement patterns over time results in the formation of indirect pathways for species introduction. The above scenario holds for biofouling as well. Species that accumulate on a ship in port *C* can still impose introduction risk to port *E* in addition to port *A*, if they manage to survive along the path *C* → *A* → E. Ignoring such dependencies will result in an inaccurate estimation of the introduction risk.

Recent research has brought to fore challenges with the first-order network view and the limitations it poses in network analysis (e.g., community detection [16–19], node ranking [20], anomaly detection [21], and representation learning [22, 23]). In the Ecology domain, recent studies have recognized the importance of higher-order dependencies in ship-borne NIS transfer and some have attempted to incorporate them into their risk models. The authors in [24, 25] propose to include the entire ship trajectory for calculating the NIS risk. However, some connections are more important than others. For example, reoccurring higher-order patterns are more likely to result in species introduction at a global scale. As a result, accumulating the risk over the entire trajectory can result in over-estimation of the risk. The authors in [8], consider shipping paths of length five and fewer to be connected. The problem with this method is that a fixed order is not realistic for all connections: as we explain further in this paper, several significant smaller or longer paths (e.g., some having up to 15th order of dependencies [26]) could exist in the network. These uniform approaches may both prevent accurate risk estimation and may also disguise likely differences between higher-order patterns of species introduction through biofouling versus ballast discharge. Lastly, previous models failed to incorporate environmental dependencies in higher-order introduction pathways, ignoring how significant higher-ordered patterns may change over time. These are important factors for forecasting NIS spread under future global change.

**Contributions**. Here, we conduct a comparative study to assess the risk of NIS spread via biofouling and ballast water through the global shipping network. To this end, we integrate the vessel movement data, environmental data, and biogeographical data to develop species-flow networks for both introduction vectors. We compare two network flow models, species flow first-order network (SF-FON) which only includes the first-order connections, and species-flow higher-order network (SF-HON) which includes all significant higher-order dependencies in the ship movement patterns. We then evaluate predictions of each model, as well as the higher-order model of [24] against NIS introduction datasets from the USA and Europe.

This study presents the first large-scale validation of higher-order NIS spread via ballast water and biofouling. Our results may inform more effective, targeted prevention strategies for NIS spread management. Our main unique contributions include:

- Providing a global model for biofouling risk by integrating the shipping, environmental and biogeographical data.
- Presenting a global comparison of the NIS spread risk via ballast water and biofouling. We identify the main differences in network structure, clustering patterns, main pathways, and illustrate the correlation between vessel characteristics and NIS spread risk for each vector.
- Quantifying the impact of incorporating higher-order dependencies into species-flow modeling by comparing the accuracy of SF-HON model against the existing higher-order approach and the SF-FON model using two large datasets of NIS from the USA and Europe.
- Illustrating changes in NIS spread risk and SF-HONs characteristics over 6 years of data shipping data spanning (1997-2012).

## 2 Materials and methods

### 2.1 Data sources

**Fig 2:**
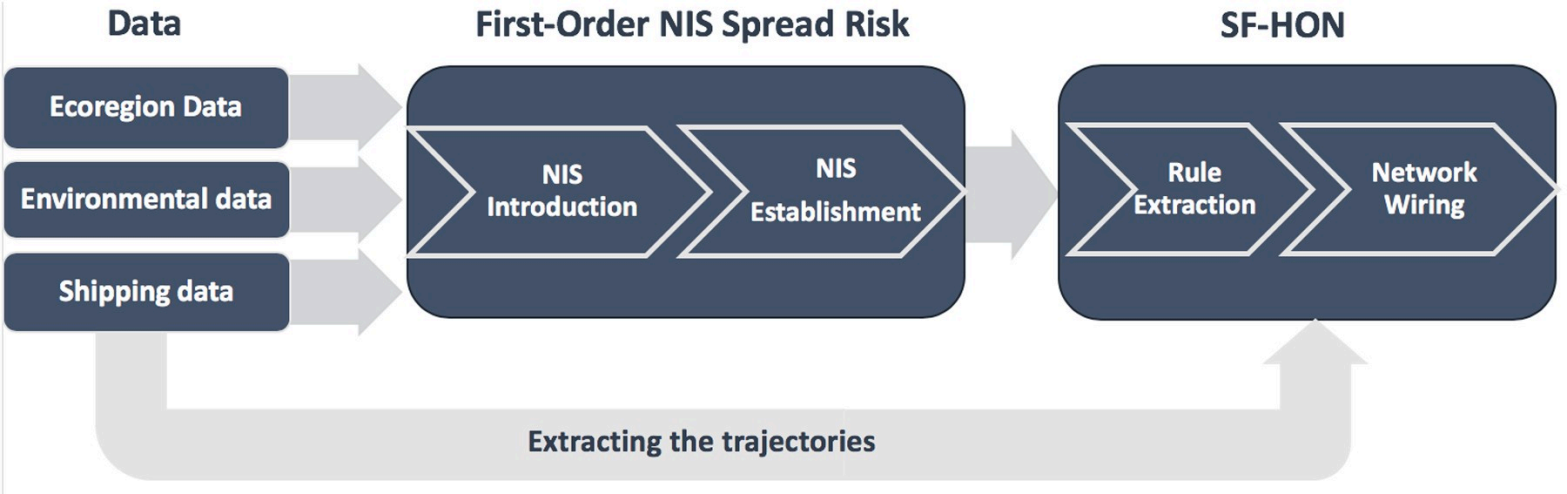
A block diagram of the risk network construction. The ecoregion data, environmental data, and the shipping data are used to compute the first-order (pairwise) NIS spread link between the shipping ports through each shipping introduction vector. The resulting probabilities along with the shipping trajectories are used for constructing the two SF-HONs, ballast SF-HON and biofouling SF-HON.

As illustrated in Fig 2, we incorporated the shipping and environmental data sources into SF-HON. We then used invasion records from *AquaNIS* data and the *NAS* data for validating the effectiveness of SF-HON. All validation datasets are filtered to include records from 1997 to the present. We assume it takes one to two decades for species to be introduced via shipping activity, thrive and reproduce in the new environment to be publicly detected as an invasive species. We have provided an aggregated view of the shipping data along with the environmental properties of the ports in this repository: http://bit.ly/2GF3fJ4.

**Ship movement data**: We obtained port locations and ship voyage data from Lloyd’s List Intelligence, an Informa Group Company (LLI, New York, NY, USA). LLI collects global port calls in conjunction with the Lloyd’s Agency Network, which gathers information from over 1200 local agents who observe port arrivals and departures directly and additionally gather data from other sources. The LLI Database is a global standard for commercial ship traffic research for government and business applications. It does not include military or recreational ships. These data contain travel information for ships including port ID, sail date and arrival date, along with ship meta-data (we used ship size, ship gross weight-tonnage, and type of the cargo carried by ship). This information is used to build both SF-FONs and SF-HONs to represent the NIS risk through ballast discharge and biofouling among ports. Our study is based on LLI data over the most recent year of data available to us starting May 2012 with 1, 185, 510 individual voyages. However, for section 3.3 (Evolution of SF-HONs and NIS risk) we used 6 years of data (spanning over 15 years) starting 1st of May 1997, with 9, 482, 285 voyages.

**Ballast discharge data**: This data is provided by the National Ballast Information Clearinghouse (NBIC) [27], and contains the date and the discharge volume of all ships visiting U.S. ports from Jan. 2012 to present. We used the NBIC data to estimate an average ballast discharge for ships given their type and size. We did not consider ballast-water exchange or other ballast water management practices that were being practiced in some jurisdictions during some of our time series.

**Environmental data**: The temperature and salinity of the shipping ports is extracted from the Global Ports Database [8] and the World Ocean Atlas [28] using the approach in [8]. From 9177 ports in the ship movement data, 6695 ports with the temperature and salinity information were included. We did not consider the salinity and temperature profiles of each voyage, which could influence biofouling survival.

**Biogeographical data**: Ecoregions are geographical regions of the ocean that share species sets due to shared evolutionary history. We obtained the ecoregion data from Marine Ecoregion of the World (MEOW) [29] to determine the likelihood of a transported species being nonindigenous given the source and destination ecoregion.

**AquaNIS nonindigenous species data**: This data set contains information on NIS introduction histories, recipient regions, taxonomy, biological traits, and impacts. This data set is frequently updated with new records and is regarded as best available [30]. The NIS data is available for species introduced to European countries and is filtered to include only species suspected to have been introduced by shipping activity (ballast water and/or biofouling) from 1997 to 2017.

**NAS nonindigenous species data**: This dataset contains reliable information about the distribution and presence of species across the United States. Data fields include information about the introduced species such as taxonomy, state, freshwater/marine. Because most of the species in this dataset are freshwater species, we used only the first introductions for each NIS in each state that were potentially transported by shipping activity (ballast water and/or biofouling) and recorded between 1997 to 2017. This criterion automatically removes all the inland states from our analysis.

**National Exotic Marine and Estuarine Species Information System (NEMESIS)**: This is the nonindigenous marine and estuarine species database for North America. Taxonomic coverage includes algae and animal species. Data fields include taxonomic descriptions and classification, global distribution, invasion history, ecological and life-history traits, and known environmental and economic impacts. We extracted the first introduction for each NIS in each state that was potentially transported by shipping activity (ballast water and/or biofouling) and recorded between 1997 to present. Similar to the NAS data, the NEMESIS data does not include any inland states in our analysis. For all three species data sources, we did not attempt to distinguish ballast mediated from biofouling mediated species.

### 2.2 Risk assessment method

In this section, we explain our calculation of first-order NIS risk, construction of SF-HON and calculation of higher-order risks, network analysis methods used, and the evaluation method. We first explain how the NIS risk through each introduction vector is calculated for every voyage between a pair of ports. We aggregate the risk overall voyages to calculate the network edge weights (equivalent to the NIS risk for each port pair) for our first-order model, SF-FON. In order to calculate the edge weights in our higher-order model (SF-HONs), we first identify significant higher-order dependencies or “rules” in the shipping network using the algorithm developed by [26]. We then build SF-HON by combining the higher-order patterns and the first-order NIS risk. In addition to comparing network structure and clustering patterns between ballast and biofouling SF-FONs and SF-HONs, we also tested how well each model predicted recent NIS introduction data from Europe (*AquaNIS*) and the USA (*NAS*). Below we describe each step in more detail.

#### 2.2.1 NIS spread

As illustrated in Fig 2, we incorporated the shipping, environmental, and biogeographical data sources to estimate NIS spread risk for each ship voyage as the product of three independent probabilities: probability of being nonindigenous to the destination port, probability of establishment (survival and reproduction), and probability of introduction to the destination port. NIS introduction risks for ballast water and biofouling were calculated using a different formula, yielding separate NIS spread risks for these two vectors. For both introduction mechanisms we define the NIS spread risk between port *i* and *j* to be the product of the three probabilities:

$$\begin{array}{c} P{\left(\text{NIS}\;\text{spread}\right)}_{ij}=P{\left(\text{nonindigenous}\right)}_{ij}\times P{\left(\text{establish}\right)}_{ij}\times P{\left(\text{intro}\right)}_{ij} \end{array}$$ (1)

**The probability of being nonindigenous** We identified port pairs that may contain nonindigenous species based on the ecoregion data, to account for the fact that many wide-spread species are found in more than one ecoregion [31], [29]. As a result, neighboring ecosystems are likely to share more species that are considered native [31].

Therefore, for source and destination ports belonging to the same or neighboring ecoregions, we define the probability that they introduce a nonindigenous species to each other to be 0; otherwise, this probability is set to 1.

$$\begin{array}{c} P{\left(\text{nonindigenous}\right)}_{ij}=\{\begin{array}{cc} 0, & \text{i}\;\&\text{j}\;\in \;\text{same}\;\text{or}\;\text{neighboring}\;\text{ecoregions}\\ 1, & \text{i}\;\&\text{j}\;\in \;\text{different}\;\text{ecoregions} \end{array} \end{array}$$ (2)

**The probability of establishment** This probability is calculated based on the environmental similarity of the give ports. We adapt the state-of-the-art formulation and parameters based on [24]: This probability is modeled as a Gaussian distribution of the temperature difference Δ*T*_*ij*_ and salinity difference Δ*S*_*ij*_ of the source and destination ports, normalized by their standard deviation, *δ*_*T*_ and *δ*_*S*_,

$$\begin{array}{c} P{\left(\text{establish}\right)}_{ij}=\alpha e^{-\frac{1}{2}\left[{\left(\frac{\Delta T_{ij})}{\delta T}\right)}^{2}+{\left(\frac{\Delta S_{ij}}{\delta S}\right)}^{2}\right]} \end{array}$$ (3)

in which *α* = 0.00015, *δ*_*T*_ = 2°*C* and *δ*_*S*_ = 10 *ppt* are chosen based on [24].

**The introduction probability via biofouling** Biofouling risk is calculated based on two main factors: species accumulation and species survival during the trip. We estimate the relative biofouling accumulation on a ship based on two parameters: the duration of stay at the source port *i*, *d*_*i*_, and the antifouling practice based on the ship type *A*^(*t*)^. The relationship between biofouling accumulation (measured as the proportion of maximum species richness) and duration in port *i* (measured in days) was derived from data taken from four studies that had tracked biofouling community accumulation overtime at multiple latitudes [32–35]. The relationship was modeled as a third-order polynomial, with accumulation at locations in tropical and subtropical latitudes (equator +/- 35 degrees latitude) and temperate latitudes having different patterns (S1 Fig in S1 File). The antifouling parameter *A*^(*t*)^ refers to the proportion of ships of a given type without an operational antifouling system. Estimates for each ship type were obtained from a survey of commercial ships in California [36] as follows: Container Ships: 0.19, Automobile Carriers: 0.20, Tankers: 0.30, Passenger Ships: 0.31, Bulk Carriers: 0.42, and General ships: 0.53, and all other commercial ships: 0.60.

The second main factor in the introduction risk via biofouling is the survival probability of species during the voyage, which is known to decrease with increasing voyage velocity *v*_*ij*_. Using experimental data from [37], we fit an exponential decay function to estimate survival probability as a function of *v*_*ij*_ (S2 Fig in S1 File).

Considering both species accumulation and survival probability, we estimate biofouling introduction risk for each voyage between port *i* and *j* as:

$$p_{ij}^{\left(v\right)}=A^{\left(v\right)}\left(\beta_{Tr_{1}}d_{i}^{3}-\beta_{Tr_{2}}d_{i}^{2}+\beta_{Tr_{3}}d_{i}\right)e^{\left(-\gamma v_{ij}\right)},Tropical\;$$ (4)

$$p_{ij}^{\left(v\right)}=A^{\left(v\right)}\left(\beta_{Tp_{1}}d_{i}^{3}-\beta_{Tp_{2}}d_{i}^{2}+\beta_{Tp_{3}}d_{i}\right)e^{\left(-\gamma v_{ij}\right)},Temperate$$ (5)

Where *v*_*ij*_ is the average voyage velocity from source port *i* to destination port *j* measured in kilometers per day. *γ* = 0.008 and *β*_*Tr*1_ = 1.29 × 10^−7^, *β*_*Tr*2_ = 8.316 × 10^−5^, *β*_*Tr*3_ = 0.0149 are the tropical coefficients and *β*_*Tp*1_ = 1.4 × 10^−9^, *β*_*Tp*2_ = 1.6566 × 10^−5^, *β*_*Tp*3_ = 5.193 × 10^−3^ are the temperate coefficients (refer to section 1 in supplementary materials for more details on model development and calculation details).

**The introduction probability via ballast discharge** Our method for estimating the introduction probability based on ballast discharge slightly alters the equation used by [24]. Let *v* be a ship traveling from port *i* to *j*, during the time $\Delta t_{ij}^{\left(v\right)}$. Species in the ship ballast water may die at a daily mortality rate of *μ*. Let $D_{ij}^{\left(v\right)}$, *ρ*^(*v*)^ ∈ [0, 1], and λ be the amount of ballast water discharged at the destination, the efficacy of ballast water management for the route, and the species introduction potential per volume of discharge. Then, the probability of ship *v* introducing species from port *i* to port *j* is given by [24]:

$$\begin{array}{c} p_{ij}^{\left(v\right)}=\rho^{\left(v\right)}\left(1-e^{-\lambda D_{ij}^{\left(v\right)}}\right)e^{-\mu \Delta t_{ij}^{\left(v\right)}}. \end{array}$$ (6)

The amount of ballast water $D_{ij}^{\left(v\right)}$ is estimated based on ship type and ship gross weight tonnage using the method proposed in [14]. The trip duration $\Delta t_{ij}^{\left(v\right)}$ is extracted from Lloyd’s data set. *μ* = 0.02 and λ = 3.22 × 10^−6^ are chosen based on [14]. Note that, our vessel movement data does not include details about partial ballast discharge between the ports. As a result, we used the total estimated volume of ballast water discharged in the intermediate ports to calculate the risk in ballast SF-HON model. This is a limitation of our study and can be improved in future work with the availability of more accurate data. Incorporating the partial ballast discharge information in our model may reduce the NIS risk in the SF-HON model.

**SF-FON**: The edges in SF-FON are equivalent to the first-order NIS introduction risk between pairs of ports. In order to calculate this value for each pair of ports (given an introduction vector) we aggregate the NIS risk of all voyages from port *i* to port *j*:

$$\begin{array}{c} P{\left(\text{intro}\right)}_{ij}=1-\prod_{r,v}\left(1-p_{ij}^{\left(v\right)}\right), \end{array}$$ (7)

where the product is taken over all routes *r* such that a ship *v* travels from port *i* to *j*.

### 2.3 SF-HON: Higher-order network of species flow risk

We calculate the NIS spread risk for every pair of ports under ballast discharge and biofouling mechanisms using the model defined in Section 2.2. At this point, we could conventionally model this data into a network structure where nodes represent the shipping ports and edges correspond to the first-order port risks. However, this interpretation is an over-simplification of the complex system of global shipping traffic. Studies show that a ship’s next port to visit can depend on previously visited ports [26]. As illustrated in Fig 1(b), ships coming to port *A* from port *C* are more likely to visit port *E* than *F*, and ships coming to port *A* from port *B* are more likely to visit port *F*, than port *E*. These patterns indicate a second-order dependency, which implies that species native to port C have a higher introduction probability to port E (via port A) and species native to port B have a higher introduction probability to port F (via port A, as illustrated in Fig 1(a)). However, if the first-order movement pattern is assumed (Fig 1(c) left), the ship has equal probabilities of visiting either port *E* or *F*. Considering only first-order interactions does not capture such introduction probabilities. Therefore, a more accurate way of wiring the shipping ports is required to account for these hidden patterns. The Higher-Order Network (HON) representation is shown in Fig 1(c) right, in which probability of going to port *E* and *F* from port *A* varies given the port visited before port *A* (i.e., probability of going from *A*|*C* to *F* is higher than probability of going from *A*|*C* to *E*). Such higher-order patterns are also very important in the environmental conditions of the ports. For example, species that are intolerant to cold temperature will probably die in port *P*_2_ if the ship travels a path of *P*_1_(*tropical*) → *P*_2_(*arctic*) → *P*_3_(*tropical*). Therefore, considering only first-order interaction of *P*_1_(*tropical*) and *P*_3_(*tropical*) does not represent the intermediate port *P*_2_(*arctic*) and its environmental conditions.

Xu et al., [26] proposed a framework to model such higher-order movement patterns in a network structure. Here we adopt this framework to assess the higher-order NIS spread risk. From a total of 1,185,510 voyages during 2012-2013 (latest available year of shipping data), we extracted 29,788 and 49,895 unique first-order pathways (pairwise connections without considering intermediate ports). For each pathway, we calculate the pairwise biofouling and ballast discharge risks between source and destination ports based on the available data (ecoregion data, environmental data, and the shipping data). We also construct the ship trajectories by listing the sequence of ports visited by each ship during a one year period. Then, we combine the pairwise risks together with the ship trajectories to determine which indirect shipping routes exhibit higher-order species introduction patterns and produce the Species Flow Higher-Order Network (SF-HON). As a result, SF-HON contains the higher-order pathways of NIS transfer in the network structure. We use the ship trajectories and the NIS spread risk of the paths as the input for SF-HON (Fig 2). We then feed these probabilities to the Rule Extraction step to identify significant higher-order dependencies from the sequence of trajectories. The generated rules will be used to generate the SF-HON in the Network Wiring step (defined below).

**Rule extraction**: In the rule extraction step, the objective is to identify the significant higher-order introduction pathways by finding the correct order of dependency in the data. We use the similar rationale as [26]: Given a pathway of order *k*: $S=\{p_{1},p_{2},p_{3},\ldots ,p_{k}\}$, We can calculate the corresponding NIS spread probability of the path using the below formula:

$$\begin{array}{c} P_{S(NISspread)}=1-\prod_{(i,j)\in S}\left(1-p_{ij}\right), \end{array}$$ (8)

In order to identify the correct order of dependency based on the shipping trajectories, using a similar approach as [26], we check whether including a previous step *p*_0_ and extending S to $S_{new}=\left\{p_{0},p_{1},p_{2},p_{3},\ldots ,p_{k}\right\}$ (of order *k*_*new*_ = *k* + 1, with NIS spread probability of $P_{S_{new}}\left(NISspread\right)$) will significantly change the NIS spread probability of the path. If so, order *k*_*new*_ is assumed as the new order of dependency, and S will be extended to $S_{new}$. The resulting rule is $P\left(\left\{p_{0},p_{1},p_{2},p_{3},\cdots \to p_{k}\right\}\right)=P_{S_{new}}\left(NISspread\right)$.

The above method only accepts rules that are significant and have reoccurred sufficiently. Therefore, random patterns would fail to meet the dependency rule in aggregating significant higher-order connections. Furthermore, our method allows for variable order of dependencies for different paths.

**Network wiring**: In the network wiring step, the extracted rules are converted to corresponding nodes and edges in the SF-HON. For example, given a discovered rule *P*({*p*_0_, *p*_1_, *p*_2_ → *p*_3_}) = 0.5 we define the edge *p*_2_|*p*_0_, *p*_1_ → *p*_3_ with the weight 0.5. This indicates that the probability of NIS spread from *p*_2_ to *p*_3_ for ships who have visited ports *p*_0_ and then *p*_1_ before coming to *p*_2_ is 0.5. The node *p*_2_|*p*_0_, *p*_1_ is a third-order node, and *p*_3_ is a first-order node. The resulting network is called SF-HON in which a node can represent a sequence of ports, and thus several nodes in SF-HON can map to a single physical port (for example, *p*_2_|*p*_0_, *p*_1_ and *p*_2_|*p*_1_ both represent the physical port *p*_2_). We construct the SF-HON for the two shipping introduction vectors and define them as *ballast SF-HON* and *biofouling SF-HON*. We perform all the further analysis based on these two SF-HONs.

#### 2.3.1 Network characteristics

Below we provide a discretion of the network characteristics we used in Section 3.1.

**Clustering Coefficient** [38] (CC). Network CC is a measure of the degree to which nodes in a graph tend to cluster together. As a global network measure, we used average CC, which is the average of the local clustering coefficients of all the nodes in the network [39]. CC for a particular node is quantifies how close its neighbours are to being a clique (complete graph). Therefore, Consider a node *v*_*i*_ in the network *G* = (*V*, *E*) with neighborhood *N*_*i*_ = {*v*_*j*_: *e*_*ij*_ ∈ *E* ∨ *e*_*ji*_ ∈ *E*}. [38] define *C*_*i*_, the CC of node *v*_*i*_, as the ratio of edges between the nodes within neighbourhood *N*_*i*_ divided by the number of edges that could possibly exist between them; i.e.,

$$\begin{array}{c} C_{i}=\frac{\left|\{e_{jk}:v_{j},v_{k}\in N_{i},e_{jk}\in E\}\right|}{k_{i}\left(k_{i}-1\right)} \end{array}$$ (9)

**Density**: The density *D* of a network *G* = (*V*, *E*) is defined as a ratio of the number of edges to the number of possible edges in a network; i.e.,

$$\begin{array}{c} D=\frac{\left|E\right|}{\frac{\left(|V|\times |V|\right)}{2}} \end{array}$$ (10)

**Betweenness centrality** [40]: Betweenness centrality of node is a measure of is a way of detecting the amount of the node centrality and as a result, the influence a node has over the flow of information in a network [41]. To compute betweenness for a node *v*_*i*_, we calculate the fraction of shortest paths between any pairs of nodes (*s*, *t*) that include *v*_*i*_; i.e.,

$$\begin{array}{c} C_{B}\left(v_{i}\right)=\sum_{s\neq v\neq t}\frac{\sigma_{st}\left(v\right)}{\sigma_{st}} \end{array}$$ (11)

where *σ*_*st*_ is the total number of shortest paths from node *s* to *t*. We calculate the shortest paths by inverting transfer probabilities between nodes. To account of the size of the graph, we normalize *C*_*B*_(*v*_*i*_) by dividing *C*_*B*_(*v*_*i*_) by its maximum value, $\frac{1}{2}\left(n-1\right)\left(n-2\right)$. This normalized measure is called relative betweenness centrality. We then use Freeman’s definition [40] for calculating the betweenness centrality of the entire graph:

$$\begin{array}{c} C_{B}\left(G\right)=\frac{2\sum_{i=1}^{n}\left[C_{B}^{\prime }\left(v^{*}\right)-C_{B}^{\prime }\left(v_{i}\right)\right]}{\left(n-1\right)} \end{array}$$ (12)

Where $C_{B}^{\prime }\left(v^{*}\right)$ is the largest value of $C_{B}^{\prime }\left(v_{i}\right)$ for any vertex *v*_*i*_ in the graph.

**Connected components**: In a strongly connected component of a directed network, any nodes can reach any other node via a directed path. In a strongly connected component, any nodes can reach any other node via a path, regardless of the direction.

#### 2.3.2 Network clustering analysis

Clustering allows us to obtain a large-scale view of NIS spread and identify the groups of ports that have a similar probability of introducing species among each other. Ports within the same cluster are relatively more connected and have a stronger chance of mutual species spread. Note that, using the higher-order approach, each physical port can belong to several clusters, because its corresponding higher-order nodes belong to different clusters. For example, *p*_2_|*p*_0_, *p*_1_ belongs to cluster 1 and *p*_2_|*p*_1_ belongs to cluster 2, while they both represent the physical node *p*_2_.

We used *Infomap* [42] as a network clustering method on the two risk networks. The basic principle of Infomap clustering is to find groups of nodes among which the species flow is quick and easy, which can be aggregated as a separate cluster. This clustering method is suitable for extracting modules of species flow since it identifies clusters by optimizing the entropy corresponding to intra-cluster and inter-clusters using a recursive random-walk method, and random walks are the most similar to the species flow pattern.

#### 2.3.3 Evaluation metrics

Our goal is to test which model is more consistent with existing data on NIS occurrence. We compare the results from SF-HONs with the conventional SF-FON and another higher-order model proposed by [24]. In [24], the authors include the entire ship trajectory for calculating the NIS risk, without any further constraints on the significance of the higher-order patterns. We refer to the baselines using this method as *Ballast All-Paths* and *Biofouling All-Paths*.

We evaluate the SF-HON predictions against SF-FON and All-Paths baselines using one dataset for European countries (the AquaNIS data) and two datasets for the United States (the NAS and NEMESIS data). We filtered all three datasets to include only species suspected to have been introduced by shipping activity from 1997 (the first year of our shipping data) to the present. However, since the introduction vector for each species record was not available, we were not able to generate separate datasets for ballast water and biofouling.

For the United States (US), we obtained a total of 251 species introduction records from NAS data and 250 species introduction records from NEMESIS data. We define the *NIS introductions* to be the normalized count (between 0 and 1) of the first introduction for each NIS reported from each state. For AquaNIs data, we extracted a total of 815 introductions for 20 European countries, and define the *NIS introductions* to be the normalized count (between 0 and 1) of the first introduction for each NIS reported from each country. Normalization is done so that we can compare the relative NIS risk across different models and different datasets.

Since the NIS introduction records (AquaNIS, NAS, and NEMESIS) are at the level of states/countries, we calculate the NIS risk for each state/country by averaging the NIS risk (using SF-HONs, SF-FONs, and All-Paths models) over all the ports corresponding to each state (and each country, in case of Europe). We define the Mean Square Error (MSE) for each model by calculating the square difference between the *NIS introductions* and the scaled NIS spread risk obtained from the network models. We report the MSE value for each model (for detailed results on each state and country, refer to S8 Fig in S1 File). Note that, we also tried using median and simple aggregation (similar to Eq 7) over all the ports corresponding to a state/country. However, aggregating the risk resulted in many countries with a risk of 1, and using the median resulted in many countries with a risk of close to zero. As a result, we chose the average value of all the ports to represent the risk of a country/state.

## 3 Results

Below we provide a comparison of the SF-HONs and SF-FONs in terms of basic network characteristics. We then compare ballast and biofouling SF-HONs in terms of species flow across major biogeographic realms, the evolution of global species flow patterns from 1997-2012, and global clustering patterns. We end by comparing the relative risk estimated by SF-HON, SF-FON and the All-Paths model (based on [24]) on large NIS introduction datasets from the USA and Europe. We leave for future modeling the estimation of reduced relative risk from interventions such as required by the BWM or by local jurisdictions.

### 3.1 Network characteristics

We first evaluate the basic network properties of the two SF-HONs (ballast and biofouling) and compare them with their first-order counterparts (SF-FONs). Our analysis indicates that ballast SF-HON includes more higher-order nodes and NIS spread pathways than biofouling SF-HON (Table 1). This result can be recognized as a function of containing potentially invasive species in ballast tanks during ship voyages compared with the hull-attached species exposed during ship transits to wave and other forces (and/or the presences of antifouling hull coatings designed to improve ship fuel economies).

**Table 1:** Comparison of basic properties of the four networks. CC refers to the connected components of the graph.

	Risk Network Models			
Network properties	Ballast SF-FON	Ballast SF-HON	Biofouling SF-FON	Biofouling SF-HON
Number of nodes	2,336	90,087	2,017	20,967
Number of edges	41,400	251,718	20,510	84,904
Avg degree	17.72	2.79	10.16	4.04
Avg clustering coefficient	0.2410	0.0128	0.1956	0.0522
Density	0.0118	0.0007	0.0081	0.0003
Num of weakly CC	1	1,107	5	104
Num of strongly CC	530	64,789	596	13,915
Avg betweenness Centrality	0.2172	0.0424	0.1592	0.0388

If no higher-order dependencies exist in the risk network, the SF-HON and SF-FON would be similar. However, this is not the case for ballast or biofouling SF-HON. Table 1 summarizes the properties of the two risk networks. The ballast SF-HON expands significantly (relative to the SF-FON) due to the inclusion of significant higher-order patterns: the number of nodes and edges grow 37.56 and 5.08 times larger than SF-FON, respectively. This increase is not as large in the biofouling SF-HON: the number of nodes and edges in SF-HON becomes 9.39 and 3.13 more than SF-FON, respectively. This indicates that there are significantly more higher-order pathways of species transfer via ballast discharge compared to biofouling. For example, species are likely to get introduced from *Singapore* to *Busan* via path: *Singapore* → *Shanghai* → *Tokyo* → *Busan*, while in biofouling SF-HON species mostly get introduced via first-order paths, (e.g. *Singapore* → *Shanghai*, or *Tokyo* → *Busan*). However, network density and network average clustering coefficient [38] is overall higher in ballast SF-HON compared to biofouling SF-HON. We observe the same pattern between ballast SF-FON and biofouling SF-FON. Furthermore, nodes in ballast networks have an overall higher betweenness centrality [40] compared to biofouling networks (both SF-HON and SF-FON). For more detailed compassion of network degree distribution, and clustering coefficient refer to S3 Fig in S1 File.

**Fig 3:**
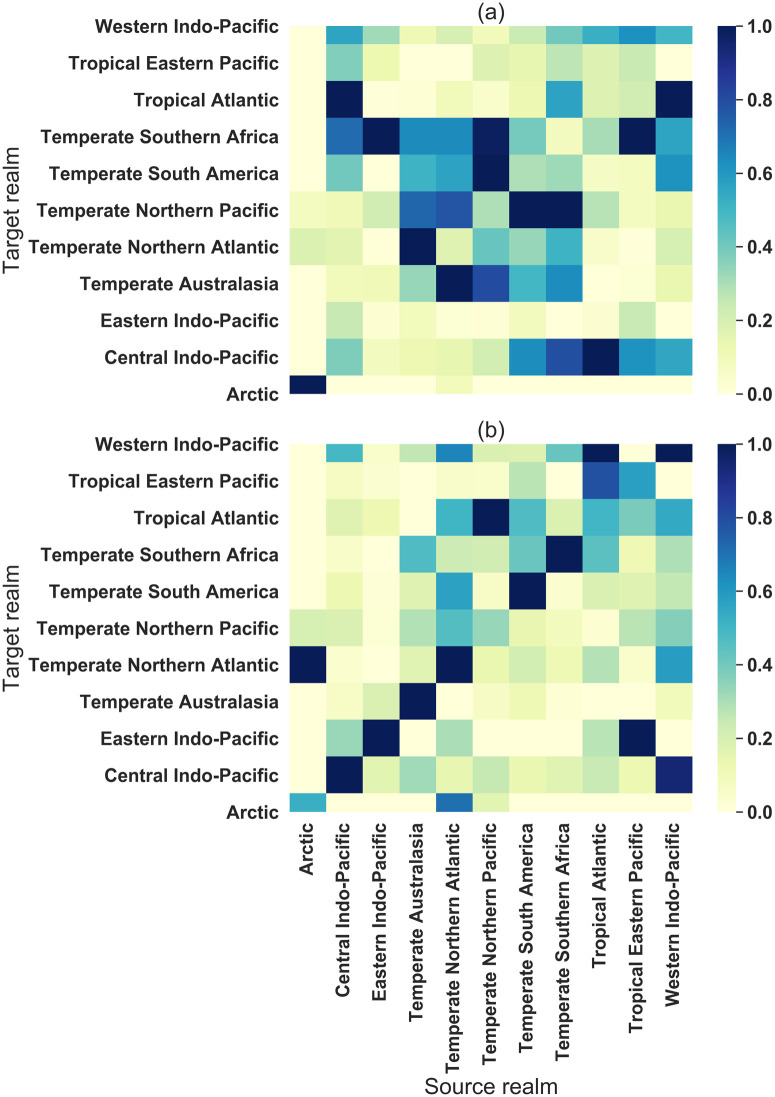
A heat-map of higher-order species flow resulting from ballast water (a) and biofouling (b) across major realms (a larger geographic scale that the ecoregion scale underlying other analyses). The y-axis indicates target realms and the x-axis are the source realms (x-axis is shared). The risks are normalized to be in the range of [0, 1]. The inter-realm risk is overall higher in ballast SF-HON (a), while most high-risk connections in biofouling SF-HON are within the same realms (b). Eastern-Indo Pacific has the highest risk of NIS introduction to Temp Southern Africa in ballast SF-HON (a), while in biofouling SF-HON the highest target realm for Eastern-Indo Pacific is Central Indo-Pacific (b). Temperate Northern Atlantic has a significantly high risk of introduction to Eastern-Indo Pacific in biofouling SF-HON (b).

These differences between the ballast and biofouling SF-HONs have implications for global NIS spread patterns. To compare large-scale patterns of species flow in both SF-HONs, we calculated the NIS spread risks between realms (large-scale biogeographical regions) by averaging over all the ports within a realm. We visualize the results as two heatmaps for ballast SF-HON (Fig 3(a)) and biofouling SF-HON (Fig 3(b)). We notice that in ballast SF-HON, realms are more likely to introduce species to each other, while in biofouling SF-HON the main high-risk connections are intra-realm ones. The main high-risk inter-realm connections in biofouling SF-HON are *Temperate Northern Pacific → Tropical Atlantic*, *Tropical Atlantic → Western Indo-Pacific*, *Tropical Eastern Pacific → Eastern Indo-Pacific*, *Western Indo-Pacific → Central Indo-Pacific*, and *Arctic → Temperate Northern Atlantic* (Fig 3(b)). This provides an overall index of the inter-realm NIS spread risk for global management plans. For a breakdown of dependency orders across major realms refer to S5 Fig in S1 File.

### 3.2 Clustering of ballast and biofouling SF-HONs

Clustering allows us to identify partitions of the networks in which ports are tightly coupled by species flow. It also provides a high-level view of interactions of the clusters. Major differences of clustering exist between the ballast and biofouling SF-HONs in terms of geographical organization of clusters, cluster overlap, and distribution of the ports within each cluster. The top 5 clusters in biofouling SF-HON cover 16.49% of all ports, while this number is 12.85% for ballast SF-HON.

**Fig 4:**
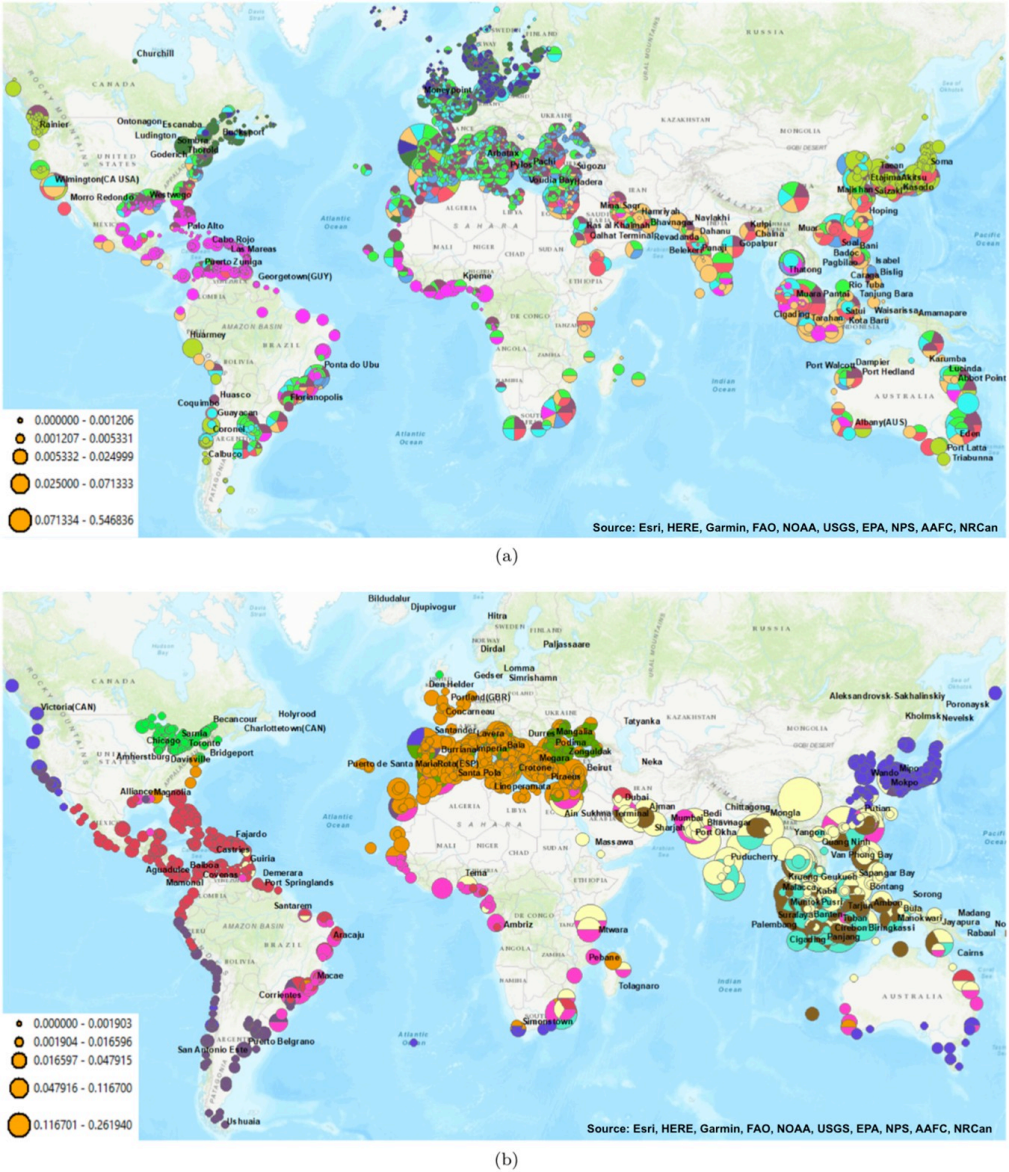
The diverse higher-order clustering of shipping ports based on the ballast discharge risks (a) and higher-order clustering of biofouling risks (b). The coloring of the ports indicates the port cluster. Ports which belong to multiple clusters through the higher-order patterns are shown as a pie chart with multiple colors. The pie chart size indicates the relative NIS spread risk for the port. The top ports with the highest risk are labeled by name.

Even though the same shipping data is used in both networks, Fig 4(a) shows that small, overlapping, and globally connected clusters predominate in the ballast SF-HON (Fig 4(a)) while larger, more isolated clusters characterize the biofouling SF-HON (Fig 4(b)).

Furthermore, Fig 4 shows that ballast SF-HON contains significantly more higher-order ports than biofouling SF-HON. Specifically, only 8.64% of the ports in the biofouling SF-HON belong to more than one cluster, while 65.14% of the ports in the ballast SF-HON belong to multiple clusters, which implies that they are more likely to introduce NIS to each other.

Overall, for the ballast SF-HON, the European cluster, the Southeast Asian cluster, and the Indian cluster contain the largest portion of higher-order nodes (Fig 4(a)). In biofouling SF-HON, only a few clusters contain significant higher-order nodes, namely, the Southeast Asian cluster, the Indian cluster, and the North African cluster (Fig 4(b)). Ports with the highest NIS spread risk and the number of clusters that they belong to are also different between the two networks (Table 2). For example, only 5 ports rank among the top 12 high-risk ports in both the ballast SF-HON and biofouling SF-HON. Additionally, the top 12 high-risk ports in the ballast SF-HON belong to 59-191 clusters, while those in the biofouling SF-HON belong in only 13-80 clusters. T2 2 lists the highest risk ports for ballast and for biofouling.

**Table 2:** Percentage of each ship type in the data for calculation of ballast and biofouling risk. Percentage values for ship types with an overall higher NIS introduction risk (as identified in Fig 8) are marked as bold. We notice that most ship types with higher introduction risk (via ballast or biofouling) do not make a large portion of the records.

	Auto	Bulk	General	Other	Ref-Cargo	Research	Chemical	Container	Gas	Passenger	Oil	Fishing	Yacht
Ballast risk	**7.92**	**22.0**	19.37	0.58	2.29	0.05	5.99	31.76	2.39	**2.01**	**8.53**	0.09	0.03
Biofouling risk	4.23	24.83	23.49	**0.82**	2.43	**0.13**	8.96	23.49	3.14	0.34	11.86	**0.17**	**0.08**

### 3.3 Evolution of SF-HONs and NIS spread risk

**Fig 5:**
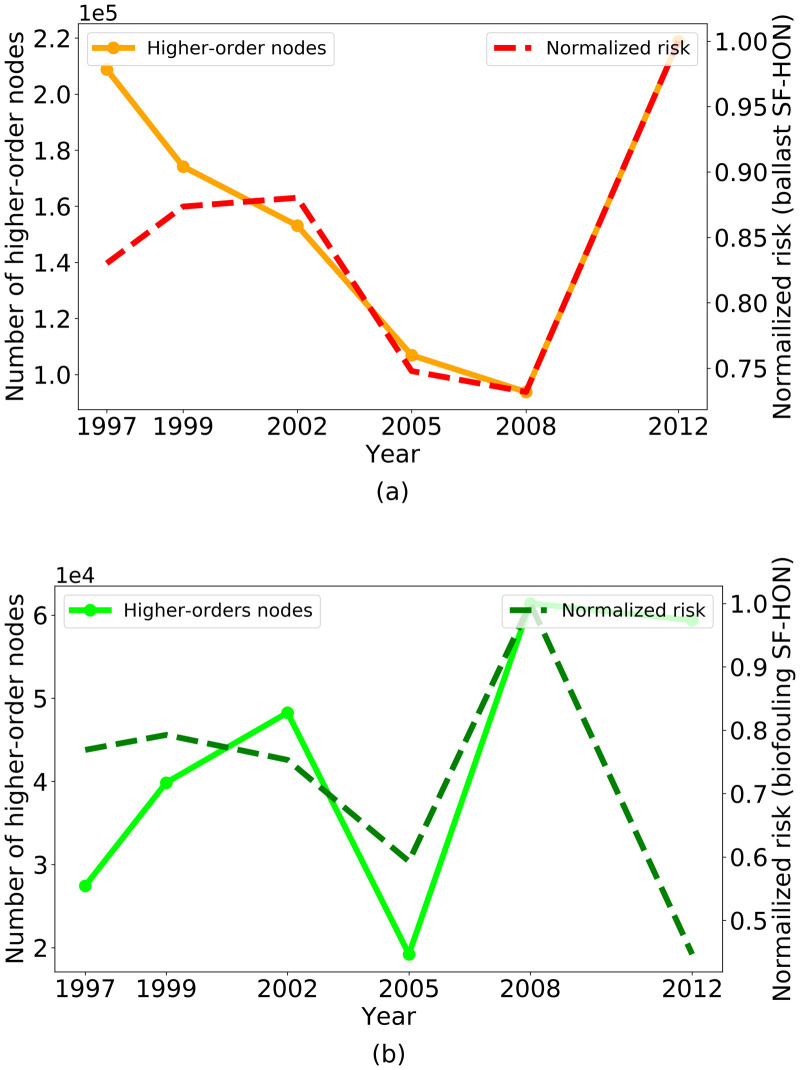
Variation of number of higher-order nodes and average NIS spread risk for (a) ballast SF-HON and (b) biofouling SF-HON. The average risk in both cases is normalized for easier comparison. In 2008 the average risk and number of higher-order nodes in ballast SF-HON drops, while they both increase in biofouling SF-HON.

**Fig 6:**
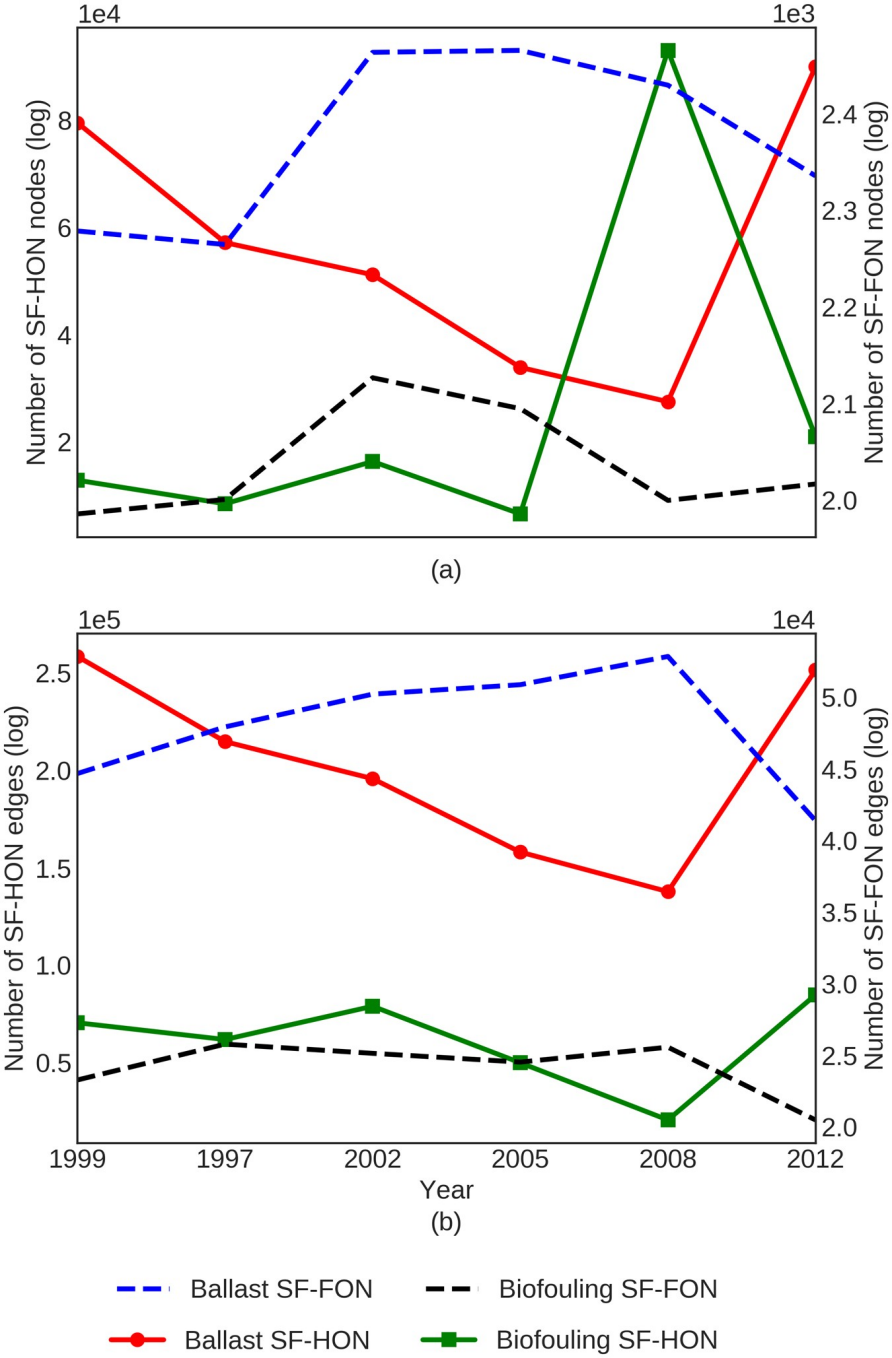
SF-HONs show significantly larger changes in both number of nodes (a) and edges (b) compared to SF-FONs.

Shipping activity, ship characteristics, trade patterns, and even environmental conditions evolve over time, and the risk of species invasions would change as these factors change. In this section, we employ the entire shipping data available to us (six years of data spanning 1997-2012) to analyze changes in network structure and the NIS spread risk for ballast and biofouling SF-HONs. In both networks, the average NIS spread risk is highly correlated with the number of higher-order nodes in the network over time (Pearson correlation value: 0.8362 (*p*-value < 10^−5^)). This indicates that higher-order nodes generally have a higher risk of NIS spread. Large changes in risk occurred over time, however. For example, in 2008 the number of higher-order nodes and average risk declined in the ballast SF-HON while they both reached their peak in the biofouling SF-HON (Fig 5). Furthermore, Fig 6(a) shows that while the number of nodes in ballast and biofouling SF-FONs change similarly over time, the number of nodes in ballast SF-HON and biofouling SF-HON change in opposing directions. Moreover, Fig 6(b) shows that the number of edges in SF-HONs change in the opposite direction of SF-FONs. Both observations demonstrate the intrinsic differences in SF-HON and SF-FON as a result of higher-order patterns. For more details about changes in network density over time refer to S4 Fig in S1 File.

### 3.4 Evaluation of SF-HON and SF-FON predictions

**Fig 7:**
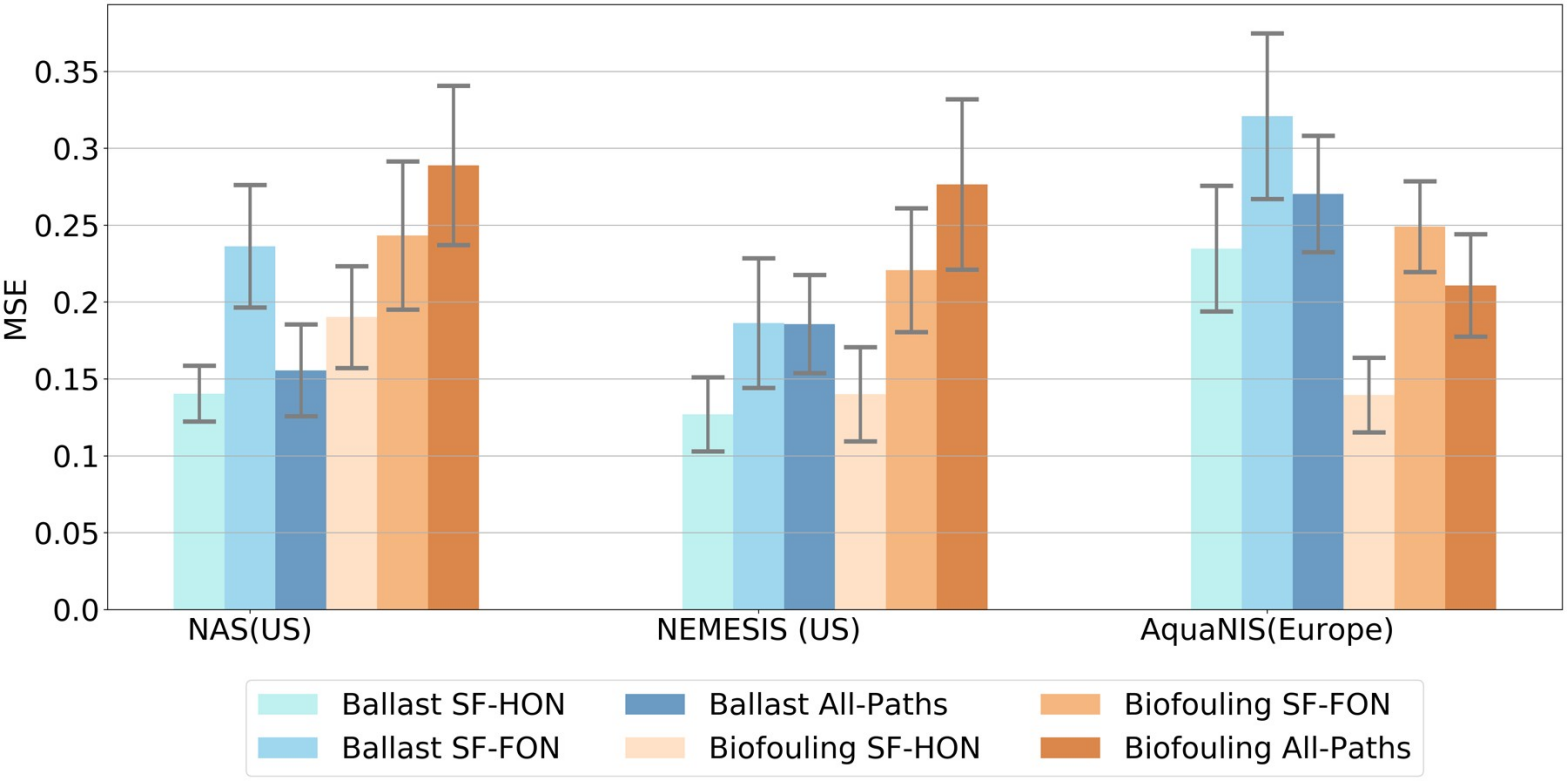
Validation results on the three data sets available. Comparison of the SF-HONs versus SF-FONs and All-Paths models shows that SF-HONs have generally ballast SF-HON yields lower MSE (suggesting more accurate results) on the NAS and NEMESIS datasets, while biofouling SF-HON yields lower MSE (more accurate results) on the AquaNIS data.

To evaluate the accuracy of the ballast and biofouling SF-HONs against the first-order and All-Paths models [25], we tested their predictions on real NIS introduction data. For each model, we calculated the Mean Square Error (MSE) with respect to previously reported NIS introductions to the USA and Europe (Fig 7). We performed a two-tailed *t*-test to evaluate the statistical significance of the errors between SF-HON and other models. For ballast NIS predictions, although SF-HON yielded the lowest MSE for all three datasets, we did not find the difference in predictions of SF-HON and the two other models to be statistically significant in all datasets (refer to S2 Table in S1 File for the *p*-values). For biofouling predictions, however, SF-HONs predictions were statistically more accurate than both SF-FON and All-Paths models in AquaNIS and NEMESIS datasets (S2 Table in S1 File). In NAS data, the difference between SF-HON and All-Paths predictions is statistically significant (*p*-value = 0.036), but for SF-HON and SF-FON the difference was not significant (*p*-value = 0.147). Overall, biofouling SF-HON improves the predictions of NIS spread significantly compared to All-paths and SF-FON. While ballast SF-HON’s predictions on average results in more accurate predictions, the improvement is not statistically significant, except on the NAS data (between SF-HON and SF-FON) and the NEMESIS data (between SF-HON and All-Paths).

We investigated the performance of each model further by looking at individual country/state predictions for all models. Both All-Paths models over-estimated the risk in 76% of all cases. The first-order models, however, under-estimated the risk in 80% of all cases (S7 Fig in S1 File).

We performed the same analysis over other years as well. SF-HONs show an overall better performance than both first-order and All-Paths models, in particular for biofouling NIS spread risk. We provided a map of high-risk introduction pathways in Europe based on biofouling SF-HON and main introduction pathways in North America based on ballast SF-HON in S6 Fig in S1 File.

### 3.5 Analysis of different ship types

**Fig 8:**
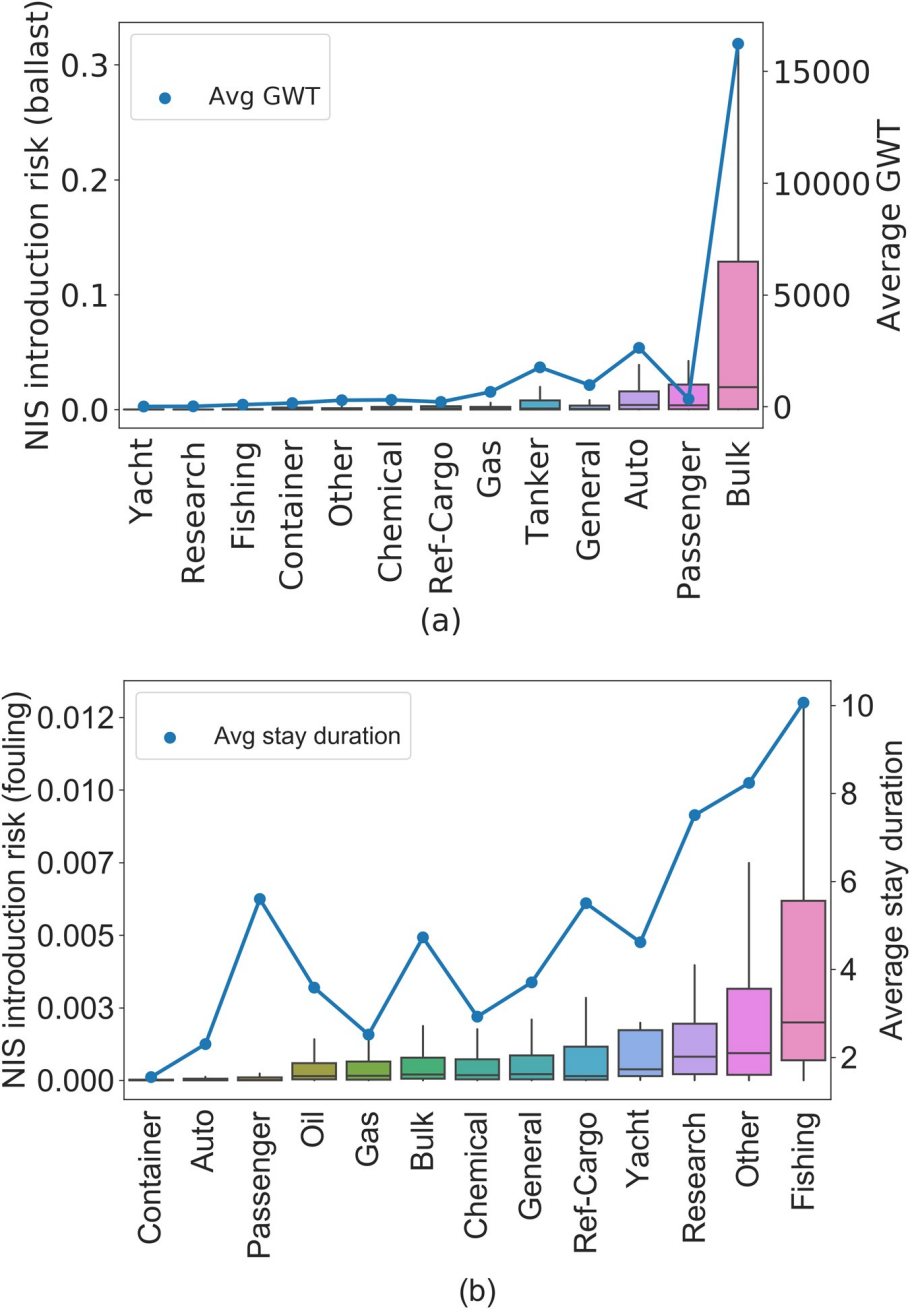
Box plots represent the NIS introduction risk for all voyages by different ship types for ballast water (a) and biofouling (b). For ballast water, GWT and for biofouling, duration of stay at port display the same pattern as the NIS introduction risk.

Given the observation that SF-HONs provide a better estimate of NIS spread risk (section 3.4), we analyze individual voyages –regardless of the shipping traffic of the ports—to understand how different ship types contribute to ballast and biofouling NIS introduction risk. Bulk and Auto carriers contribute the greatest risk for NIS introductions through ballast water (Fig 8(a)). However, looking at Table 2 we can see that Bulk and Auto carries are only 22% and 7.92% of the records. On the other hand, the percentage of the records for Container (31.76%) and General (19.37%) carriers is about the same or even higher (Table 2), but the ballast NIS risk for these ships are significantly lower than Bulk and Auto carries (Fig 8(a)). The reason is directly related to ship Gross Weight Tonnage (GWT), which is a good measure of ship size. Container and General carriers have a significantly smaller GWT compared to Bulk, Auto and Passenger carriers (Fig 8(a)).

For biofouling risk, many different carriers are responsible for introduction (Fig 8(b)). In particular, Fishing, Other, Research, and Yacht carriers are the main contributors to biofouling introduction. Again, Table 2 indicates that these main contributors are not a large portion of all the risk records. Fishing carriers are only (0.17%) of the records. Research (0.13%), Yacht (0.08%), and Other (0.82%) carriers each consist of a small portion of all the records while Container (23.49%), Bulk (24.83%), and General (23.49%) carriers are the major portion of the data. In this case, we realized that based on our model, the average duration of stay at source port is a key factor in biofouling risk. Fig 8(b) shows that ship types with higher average stay duration have a higher NIS risk through biofouling.).

Recall that biofouling clusters are more localized. Many high-risk ports are distributed across these clusters. Fishing, Research, Yacht, (and possibly the “Other” category) correspond to several small ships traveling through shorter distances which do not carry a large amount of cargo and have fewer regulations imposed on them for using an antifouling system [36]. As a result, these local ships can easily transfer species locally within the small clusters in Fig 4(b).

## 4 Discussion

We integrated global spatial patterns of ship voyages, characteristics of different ship types that affect species vectoring, aquatic environmental conditions, and biogeography into predictive models of NIS spread risk. Without the understanding provided by such models, it will be difficult to evaluate existing policies or design more effective and efficient future policies and management actions to reduce species invasions by two of the most important NIS vectors globally, ballast and biofouling. We have shown for the first time that incorporating significant higher-order patterns of the shipping network significantly improves the accuracy of predictions of NIS spread for biofouling transfer. Furthermore, with the first-ever global species flow model for biofouling, we have shown that the spatial patterns of species introductions via biofouling, which have been much less studied than ballast-mediated introductions, are likely to differ greatly from those of ballast. This has important implications for future policies and management to address biofouling. Below we discuss these points further and highlight needs for future research.

### Incorporating higher-order shipping patterns improves the accuracy of NIS risk predictions

This work advances the understanding of ship-borne invasive species risk by demonstrating that incorporating recurrent higher-order patterns into models of the shipping network improves the accuracy of predictions of global ship-born NIS spread. For all three species datasets for both ballast and biofouling models, our SF-HON outperformed SF-FON and a previously published higher-order model [24]. This improvement was statistically significant for biofouling prediction. These results strongly suggest that ship-borne species introductions conform to a higher-order process, the structure of which depends on the incomplete release of all propagules at each port, and on the specific and repeated ordering of shipping voyages (and not on every higher-order pattern). Both phenomena, incomplete propagule release and repeated patterns of ship voyages are well known (see for ballast discharge patterns in the US and [43] for global voyage patterns), but have not previously been incorporated into network models in this way. This study confirms their importance to accurate prediction of ship-borne NIS introductions globally.

Our SF-HON models, which included only significant higher-order connections, provided the most accurate results and therefore represent an improvement over previous global spread models [44, 8, 24]. We found that including the entire ship trajectories for risk calculation likely overestimated risk from longer pathways, resulting in over-estimation of the overall NIS spread risk. This result is consistent with another evaluation of the All-Paths ballast model which found that, while its accuracy in predicting the distribution of 40 NIS was very good (77%), it tended to over-predict NIS spread [25]. First-order models, on the other hand, do not consider any pathways beyond first-step connections and therefore ignore significant higher-order connections, likely under-estimated risks in most cases according to our results. While introductions from both ballast and biofouling are best characterized by Sf-HONs, the predicted spatial patterns of species introductions via these two vectors exhibit important differences from each other that must be considered to develop effective policy, which we discuss further below.

### Higher-order patterns of spread differ by vector and over time

Analyzing ballast and biofouling SF-HON models using six years of data spanning 15 years of shipping activity highlights how the temporal dynamics of the shipping network can affect the NIS spread risk in different ways through these two main shipping introduction vectors. The lengths of higher-order connections differed between ballast water and biofouling vectors, as well as within these vectors overtime during the years covered by our study. In general, the ballast SF-HON had more nodes and more edges than the biofouling SF-HON, but the numbers of nodes and edges changed over time for both ballast and biofouling SF-HONs (Fig 6). The most marked changes coincided with the global recession (2007-2009), during which a significant reduction in trade and shipping activities occurred. Specifically, during 2008, we revealed increases in average voyage distance (1.9 times more than the average), voyage duration (1.4 times longer than average), and ship stay duration at ports (4.8 times more than the average). In contrast, the number of voyages per port (60% less than the average) and edges in both ballast and biofouling SF-HONs decreased, indicating lower shipping activity. As ships spent more time at the ports, more biofouling species likely accumulated on the ship hull in greater numbers, causing modeled biofouling risk to peak in 2008. On the other hand, modeled ballast risk dropped with increased voyage duration and voyage distance, corresponding to longer connections. We hypothesize that global recession likely decreased introductions via the ballast vector and increased introductions via the biofouling vector, but without more years of data and a better sense of inter-annual variability, our confidence in this causal interpretation is limited.

Temporal changes in the shipping network over time resulted in dramatically different modeled invasion risks for the ballast and biofouling SF-HONs, highlighting the need to consider these two transport mechanisms separately when forecasting risk under different shipping scenarios. Based on the data-driven parameterization of our models, biofouling risk is highly affected by the duration of stay at the source port, while ballast transfer risk is mostly affected by the shipping traffic of the destination port. These insights can help us predict ship-born species introduction patterns for both vectors under different global socioeconomic scenarios that effect global trade and shipping, such as, for example, those currently being evaluated by the International Panel on Climate Change [45].

### Clustering of ports differs between ballast and biofouling networks

Clustering analysis of ballast and biofouling SF-HONs revealed fundamental differences in large-scale species spread patterns via each vector, which indicates the necessity of considering both vectors both separately and in tandem in order to develop effective aquatic invasions policy [10]. Species spread via ballast results in significantly more higher-order ports and more clusters than via biofouling (Fig 4). Clusters in the biofouling SF-HON are more geographically localized, with fewer inter-cluster connections. Ballast clusters, on the other hand, have a high inter-cluster connection, and are less defined by geographical proximity. Therefore, a management hypothesis to test in future work would be that targeting the inter-cluster links would likely be a more effective and efficient strategy for reducing biofouling risk than for ballast risk. Ballast management is more likely to require global prevention strategies like those required in the recently in force UN BWM agreement, but would also benefit from management interventions targeting the inter-cluster connections or major hubs receiving diverse shipping traffic (see Xu et al. 2014 for examples of network-informed policy strategies).

### Opportunities for future research

The development and evaluation of our models for the spread of species by ballast and biofouling were based on the availability of data on a global scale. Opportunities for future model improvement include: the incorporation of more complete ship data, including military [46, 47] and recreational ships [48], when such data become available; incorporation of the likely effect on biofouling risk of ambient ocean temperature and salinity along voyage routes [49, 50]; more information on the determinant of fouling in niche areas [51, 52]; and more complete data on species occurrence such as can be provided by eDNA surveys [53]. However even with existing shipping data and without all such possible improvements, opportunities for important policy-relevant advances exist by incorporating greater detail on ship-voyage specific NIS management practices including ballast water exchange, ballast water treatment, and anti-fouling practices [36, 52]. Our results particularly highlight the potential importance of future work on how alternative management strategies tailored to ship types and voyages might efficiently reduce overall invasion risk, based on the large differences in NIS risk posed by different ship types, different voyage routes, and the different characteristics of the ballast SF-HON relative to the biofouling SF-HON.

## Supporting information

S1 Appendix (TEX)

S1 File (PDF)

## References

[1] Pimentel D, Zuniga R, Morrison D. Update on the environmental and economic costs associated with alien-invasive species in the United States. Ecological economics. 2005;52(3):273–288. 10.1016/j.ecolecon.2004.10.002

[2] Molnar JL, Gamboa RL, Revenga C, Spalding MD. Assessing the global threat of invasive species to marine biodiversity. Frontiers in Ecology and the Environment. 2008;6(9):485–492. 10.1890/070064

[3] Carlton JT. The scale and ecological consequences of biological invasions in the World’s oceans. Invasive species and biodiversity management. 2001;24:195.

[4] Carlton JT. Marine bioinvasions: the alteration of marine ecosystems by nonindigenous species. Oceanography. 1996;9(1):36–43. 10.5670/oceanog.1996.25

[5] Hulme PE. Trade, transport and trouble: managing invasive species pathways in an era of globalization. Journal of applied ecology. 2009;46(1):10–18.

[6] Carlton JT, Ruiz GM. Vector science and integrated vector management in bioinvasion ecology: conceptual frameworks. SCOPE-SCIENTIFIC COMMITTEE ON PROBLEMS OF THE ENVIRONMENT INTERNATIONAL COUNCIL OF SCIENTIFIC UNIONS. 2005;63:36.

[7] Leung B, Lodge DM, Finnoff D, Shogren JF, Lewis MA, Lamberti G. An ounce of prevention or a pound of cure: bioeconomic risk analysis of invasive species. Proceedings of the Royal Society of London B: Biological Sciences. 2002;269(1508):2407–2413. 10.1098/rspb.2002.2179 PMC1691180 12495482

[8] Keller RP, Drake JM, Drew MB, Lodge DM. Linking environmental conditions and ship movements to estimate invasive species transport across the global shipping network. Diversity and Distributions. 2011;17(1):93–102. 10.1111/j.1472-4642.2010.00696.x

[9] Chan FT, MacIsaac HJ, Bailey SA. Relative importance of vessel hull fouling and ballast water as transport vectors of nonindigenous species to the Canadian Arctic. Canadian Journal of Fisheries and Aquatic Sciences. 2015;72(8):1230–1242. 10.1139/cjfas-2014-0473

[10] Williams SL, Davidson IC, Pasari JR, Ashton GV, Carlton JT, Crafton RE, et al Managing multiple vectors for marine invasions in an increasingly connected world. Bioscience. 2013;63(12):952–966. 10.1525/bio.2013.63.12.8

[11] Muirhead JR, Minton MS, Miller WA, Ruiz GM. Projected effects of the Panama Canal expansion on shipping traffic and biological invasions. Diversity and Distributions. 2015;21(1):75–87. 10.1111/ddi.12260

[12] Fofonoff PW, Ruiz GM, Steves B, Carlton JT. In ships or on ships? Mechanisms of transfer and invasion for nonnative species to the coasts of North America. Invasive species: vectors and management strategies. 2003;152:162–169.

[13] Andersen MC, Adams H, Hope B, Powell M. Risk assessment for invasive species. Risk analysis. 2004;24(4):787–793. 10.1111/j.0272-4332.2004.00478.x 15357799

[14] Xu J, Wickramarathne TL, Chawla NV, Grey EK, Steinhaeuser K, Keller RP, et al. Improving management of aquatic invasions by integrating shipping network, ecological, and environmental data: data mining for social good. In: Proceedings of the 20th ACM SIGKDD international conference on Knowledge discovery and data mining. ACM; 2014. p. 1699–1708.

[15] Hewitt CL, Gollasch S, Minchin D. The vessel as a vector–biofouling, ballast water and sediments. In: Biological invasions in marine ecosystems. Springer; 2009 p. 117–131. 10.1007/978-3-540-79236-9_6

[16] Rosvall M, Esquivel AV, Lancichinetti A, West JD, Lambiotte R. Memory in network flows and its effects on spreading dynamics and community detection. Nature communications. 2014;5 10.1038/ncomms5630 25109694

[17] Benson AR, Gleich DF, Leskovec J. Higher-order organization of complex networks. Science. 2016;353(6295):163–166. 10.1126/science.aad9029 27387949 PMC5133458

[18] Benson AR, Gleich DF, Leskovec J. Tensor spectral clustering for partitioning higher-order network structures. In: Proceedings of the 2015 SIAM International Conference on Data Mining. SIAM; 2015. p. 118–126. 10.1137/1.9781611974010.14 PMC5089081 27812399

[19] Zhou D, Zhang S, Yildirim MY, Alcorn S, Tong H, Davulcu H, et al. A local algorithm for structure-preserving graph cut. In: Proceedings of the 23rd ACM SIGKDD International Conference on Knowledge Discovery and Data Mining; 2017. p. 655–664.

[20] Scholtes I, Wider N, Garas A. Higher-order aggregate networks in the analysis of temporal networks: path structures and centralities. The European Physical Journal B. 2016;89(3):61 10.1140/epjb/e2016-60663-0

[21] Xu J, Saebi M, Ribeiro B, Kaplan LM, Chawla NV. Detecting anomalies in sequential data with higher-order networks. arXiv preprint arXiv:171209658. 2017;.

[22] Saebi M, Ciampaglia GL, Kaplan LM, Chawla NV. HONEM: Network Embedding Using Higher-Order Patterns in Sequential Data. arXiv preprint arXiv:190805387. 2019;.

[23] Rossi RA, Ahmed NK, Koh E. Higher-order Network Representation Learning. In: Companion of the The Web Conference 2018 on The Web Conference 2018. International World Wide Web Conferences Steering Committee; 2018. p. 3–4.

[24] Seebens H, Gastner M, Blasius B. The risk of marine bioinvasion caused by global shipping. Ecology letters. 2013;16(6):782–790. 10.1111/ele.12111 23611311

[25] Seebens H, Schwartz N, Schupp PJ, Blasius B. Predicting the spread of marine species introduced by global shipping. Proceedings of the National Academy of Sciences. 2016;113(20):5646–5651. 10.1073/pnas.1524427113 PMC4878520 27091983

[26] Xu J, Wickramarathne TL, Chawla NV. Representing higher-order dependencies in networks. Science advances. 2016;2(5):e1600028 10.1126/sciadv.1600028 27386539 PMC4928957

[27] Clearinghouse NBI. NBIC Online Database. Electronic publication, Smithsonian Environmental Research Center & United States Coast Guard.; 2016.

[28] Baranova O, et al. World ocean atlas 2013; 2013.

[29] Spalding MD, Fox HE, Allen GR, Davidson N, Ferdana ZA, Finlayson M, et al Marine ecoregions of the world: a bioregionalization of coastal and shelf areas. BioScience. 2007;57(7):573–583. 10.1641/B570707

[30] Board AE. Information system on Aquatic Non-Indigenous and Cryptogenic Species. World Wide Web electronic publication.; 2015.

[31] Abell R, Thieme ML, Revenga C, Bryer M, Kottelat M, Bogutskaya N, et al Freshwater ecoregions of the world: a new map of biogeographic units for freshwater biodiversity conservation. BioScience. 2008;58(5):403–414. 10.1641/B580507

[32] Schoener A, Long ER, DePalma J. Geographic variation in artificial island colonization curves. Ecology. 1978;59(2):367–382. 10.2307/1936380

[33] Canning-Clode J, Maloney KO, McMahon SM, Wahl M. Expanded view of the local–regional richness relationship by incorporating functional richness and time: a large-scale perspective. Global Ecology and Biogeography. 2010;19(6):875–885. 10.1111/j.1466-8238.2010.00560.x

[34] Canning-Clode J, Fofonoff P, Riedel GF, Torchin M, Ruiz GM. The effects of copper pollution on fouling assemblage diversity: a tropical-temperate comparison. PloS one. 2011;6(3):e18026 10.1371/journal.pone.0018026 21437262 PMC3060921

[35] Freestone AL, Ruiz GM, Torchin ME. Stronger biotic resistance in tropics relative to temperate zone: effects of predation on marine invasion dynamics. Ecology. 2013;94(6):1370–1377. 10.1890/12-1382.1 23923500

[36] Scianni C, Brown C, Newsom A, Nedelcheva R, Falkner M, Dobroski N. 2013 Biennial Report on the California Marine Invasive Species Program; 2013.

[37] Coutts AD, Piola RF, Taylor MD, Hewitt CL, Gardner JP. The effect of vessel speed on the survivorship of biofouling organisms at different hull locations. Biofouling. 2010;26(5):539–553. 10.1080/08927014.2010.492469 20526914

[38] Watts DJ, Strogatz SH. Collective dynamics of ‘small-world’networks. nature. 1998;393(6684):440 10.1038/30918 9623998

[39] Kemper A. Valuation of network effects in software markets: A complex networks approach. Springer Science Business Media; 2009.

[40] Freeman LC. A set of measures of centrality based on betweenness. Sociometry. 1977; p. 35–41. 10.2307/3033543

[41] Golbeck J. Analyzing the social web. Newnes; 2013.

[42] Rosvall M, Bergstrom CT. Maps of random walks on complex networks reveal community structure. Proceedings of the National Academy of Sciences. 2008;105(4):1118–1123. 10.1073/pnas.0706851105 PMC2234100 18216267

[43] Kaluza P, Kölzsch A, Gastner MT, Blasius B. The complex network of global cargo ship movements. Journal of the Royal Society Interface. 2010;7(48):1093–1103. 10.1098/rsif.2009.0495 PMC2880080 20086053

[44] Drake JM, Lodge DM. Global hot spots of biological invasions: evaluating options for ballast–water management. Proceedings of the Royal Society of London B: Biological Sciences. 2004;271(1539):575–580. 10.1098/rspb.2003.2629 PMC1691629 15156914

[45] Riahi K, Van Vuuren DP, Kriegler E, Edmonds J, O’neill BC, Fujimori S, et al The shared socioeconomic pathways and their energy, land use, and greenhouse gas emissions implications: an overview. Global Environmental Change. 2017;42:153–168. 10.1016/j.gloenvcha.2016.05.009

[46] Burkholder JM, Hallegraeff GM, Melia G, Cohen A, Bowers HA, Oldach DW, et al Phytoplankton and bacterial assemblages in ballast water of US military ships as a function of port of origin, voyage time, and ocean exchange practices. Harmful Algae. 2007;6(4):486–518. 10.1016/j.hal.2006.11.006

[47] Cofrancesco AF Jr, Reaves DR, Averett DE. Transfer of invasive species associated with the movement of military equipment and personnel. Engineer Research and Development Center Vicksburg MS Environmental Lab; 2007.

[48] Clarke Murray C, Pakhomov EA, Therriault TW. Recreational boating: a large unregulated vector transporting marine invasive species. Diversity and Distributions. 2011;17(6):1161–1172. 10.1111/j.1472-4642.2011.00798.x

[49] Davidson IC, McCann LD, Fofonoff PW, Sytsma MD, Ruiz GM. The potential for hull-mediated species transfers by obsolete ships on their final voyages. Diversity and Distributions. 2008;14(3):518–529. 10.1111/j.1472-4642.2008.00465.x

[50] Brock R, Bailey-Brock JH, Goody J. A case study of efficacy of freshwater immersion in controlling introduction of alien marine fouling communities: the USS Missouri. 1999;.

[51] Moser CS, Wier TP, First MR, Grant JF, Riley SC, Robbins-Wamsley SH, et al Quantifying the extent of niche areas in the global fleet of commercial ships: the potential for “super-hot spots” of biofouling. Biological Invasions. 2017;19(6):1745–1759. 10.1007/s10530-017-1386-4

[52] Davidson IC, Scianni C, Minton MS, Ruiz GM. A history of ship specialization and consequences for marine invasions, management and policy. Journal of Applied Ecology. 2018;55(4):1799–1811. 10.1111/1365-2664.13114

[53] Grey EK, Bernatchez L, Cassey P, Deiner K, Deveney M, Howland KL, et al Effects of sampling effort on biodiversity patterns estimated from environmental DNA metabarcoding surveys. Scientific Reports. 2018;8(1):1–10. 10.1038/s41598-018-27048-2 29891968 PMC5995838

