# Supplementary material for: Higher-order patterns of aquatic species spread through the global shipping network
Source: PLoS One. 2020 Jul 31;15(7):e0220353. doi: 10.1371/journal.pone.0220353 (PMC7394518; doi:10.1371/journal.pone.0220353)
Supplement: S1 File — (PDF) [file pone.0220353.s002.pdf]

Supplementary materials

1 Biofouling NIS introduction model

In this work, we develop a model for biofouling NIS introduction via commercial ship voyages based on available empirical data. Biofouling is a complex series of events that begins with the accumulation of microbial biofilms on submerged hard surfaces and is followed by the colonization of larger sedentary organisms such as algae and invertebrates and then motile organisms such as fish and decapods. Previous models of ship-mediated non-indigenous species (NIS) spread have focused on ballast water transport [1, 2, 3, 4, 5]. In comparison, fewer models have been proposed for biofouling [4, 6]), despite the fact that this vector is considered responsible for as many if not more NIS establishments [74% in Hawaii [7], 87% in New Zealand [8], 78% in Port Phillip Bay Australia [9], 50% in the North Sea [10], 70% in coastal North America [11]].

Biofouling has been studied extensively given its economic importance and its ecological significance. Here we focus on estimating two key processes relevant to the introduction of biofouling NIS via commercial ships: I) Extent of biofouling that accumulates on a ship in a source port and II) the probability that biofouling organisms survive transport from a source port to a recipient port.

I. Biofouling Accumulation in Source Port. The extent of biofouling on a ship can be characterized in two ways: the number of different species that have colonized a surface (species richness) or the extent to which these species cover available surface (percent cover). Here we focus on estimating species richness and not percent cover, since even small individuals can survive voyages and reproduce [12], and because ships with significant percent cover are likely to be noticed and cleaned. Ecological studies tend to find that biofouling species richness changes over time, usually beginning with a rapid increase in richness as organisms colonize bare space, followed by a plateau or even decline in richness as species interactions (e.g. competition, predation) come into play. Global studies of biofouling richness have found that the tropics have higher richness at the regional scale [13], although higher predation rate in the tropics decreases local richness more compared to subtropical or temperate regions [14]. On average, bare space colonization rate (the number of new species or individuals per unit time) increases with water temperature [15] and fluctuates seasonally in temperate climates but is more stable in tropical climates.

To predict biofouling accumulation on ships the data available to us (e.g., ship duration of stay in ports, ship type, port locations), we used biofouling richness data from studies that monitored coastal sites over time at temperate and tropical latitudes [15, 16, 13, 14]. In order to compare the data from these studies, which used different methods and recorded different taxonomic groups, we calculated the proportion of maximum species richness at each site for each study. We then fit 3rd-order polynomial regression models to tropical and temperate proportional richness data separately, as temperate species richness was overall lower and predicted to show different seasonal dynamics. Biofouling richness in the tropics was found to peak at 4 months ($\sim$120 days) just above 80% maximum observed richness and then declined slowly to just under 50% maximum richness over the rest of the year, until a slight uptick in the last month Figure S1. The temperate richness trend line increased more slowly to a peak of just under 50% at 7 months (210 days) followed by a slow decline to just above 35% at the end of a year. AIC model selection considering 1st-order, 2nd-order, and 3rd-order polynomial models for tropical and temperate data ranked the 3rd-order polynomial model best for tropical data.

In addition to time and source port latitude, we also considered the use of antifouling paints or other antifouling systems base on ship type for biofouling accumulation estimation. Antifouling paint reduces successful colonization and adherence of biofouling organisms on ship surfaces, and the effectiveness of the paint can vary by paint type, how thoroughly it is applied, and how long since last application. Here we did not consider antifouling paint explicitly because we did not have data on how paint type and application practices varied by ship types or locations. Other antifouling systems include electrolytic systems, chemical dosing, ultrasonic systems, and electro-chlorination and their use is much more variable

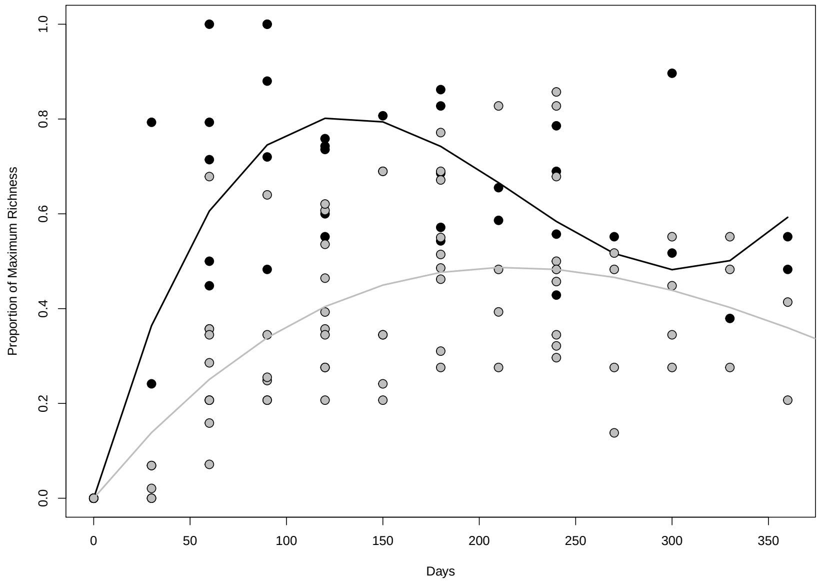

Figure S1: Biofouling richness over time, expressed as a proportion of maximal richness observed in each study by days submerged. Black circles represent tropical data points and grey circles represent temperate data points. Lines represent best fit cubic line regressions for tropical (black) and temperate (grey) data.

across ships. A survey of commercial ships in California found that the likelihood that a ship had one or more functional antifouling systems varied by ship type [17]. Based on this study, the proportion of ships with an operational antifouling system were as follows: Container Ships 0.19, Automobile Carriers 0.20, Tankers 0.30, Passenger Ships 0.31, Bulk Carriers 0.42, and "General" ships 0.53, and all other commercial ships 0.60.

Considering both the time spent in port and the likelihood of antifouling system presence, we estimate biofouling species richness on a ship at source port i as a proportion of maximum richness as follows:

$$R_i^{(v)} = A^{(t)}(\beta_{Tr_1} d_i^3 - \beta_{Tr_2} d_i^2 + \beta_{Tr_3} d_i), Tropical \tag{1}$$

$$R_i^{(v)} = A^{(t)}(\beta_{Tp_1} d_i^3 - \beta_{Tp_2} D_i^2 + \beta_{Tp_3} d_i), Temperate \tag{2}$$

Where d_i is the time (in days) that ship v stays at source port i, and $A^{(t)}$ refers to the proportion of ships of a given type without an operational antifouling system. $\beta_{Tr1} = 1.29 \times 10^{-7}$, $\beta_{Tr2} = 8.316 \times 10^{-5}$, $\beta_{Tr3} = 0.0149$ are the tropical coefficients and $\beta_{Tp1} = 1.4 \times 10^{-9}$, $\beta_{Tp2} = 1.6566 \times 10^{-5}$, $\beta_{Tp3} = 5.193 \times 10^{-3}$ are the temperate coefficients.

II. Survival to Recipient Port. Characteristics of a ship voyage can influence the survival of biofouling organisms. For example, high ship velocities cause strong sheer forces that can dislodge biofouling organisms from ship surfaces, especially if those surfaces are covered in antifouling paint. Coutts et al. [18] experiment shows a significant reduction of biofouling richness at voyage velocities greater than 14 knots. Using these results, we fit an exponential equation to estimate the decline in survival probability as a function of ship velocity as follows:

$$P_{Survival}^{(v)} = e^{-\gamma v_{ij}} \tag{3}$$

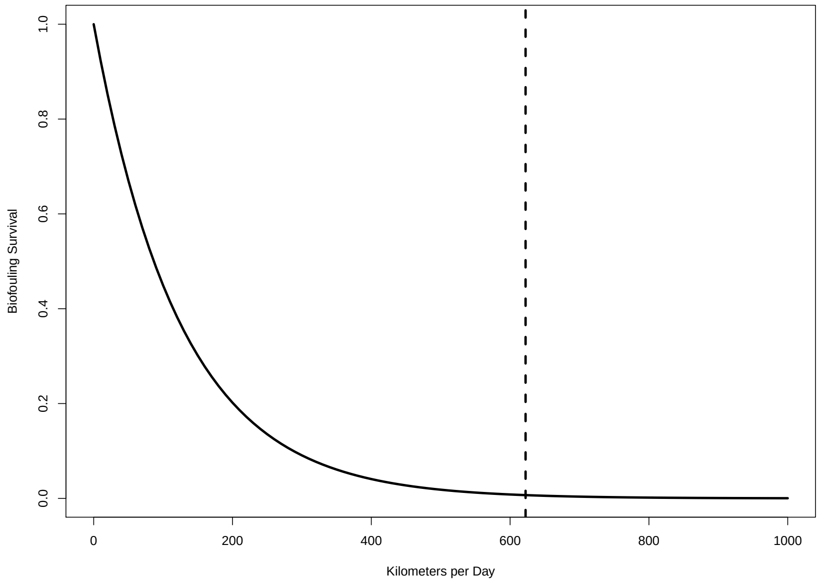

Figure S2: Biofouling survival modeled as a function of velocity (kilometers per day). The dotted line represents 14 knots, or 622.2 kilometers per day, the velocity at which survival has been found to be near zero [18].

Where v_{ij} is the average voyage velocity of ship v from source port i to destination port j measured in kilometers per day, and $\gamma = 0.008$. This model (displayed in Figure S2) yields a survival rate of 0.007 at 14 knots (or 622.2 kilometers per day), which is similar to the survival rate experimentally found in [18].

Total Voyage Introduction Risk We estimate the relative biofouling risk of each ship voyage p_{ij} in the Lloyd's dataset by multiplying equations 1 (or 2) and 3.

If | source port latitude | < 35.0 decimal degrees:

$$p_{ij}^{(v)} \sim R_i^{(v)}(Tropical) \times P_{Survival}^{(v)}$$

If | source port latitude| ≥ 35.0 decimal degrees:

$$p_{ij}^{(v)} \sim R_i^{(v)}(Temperate) \times P_{Survival}^{(v)}$$

Caveats As mentioned at the beginning of this section, biofouling is a complex process that is dependent on many environmental, ship, and voyage factors. For the purposed of our research, we focused on modeling biofouling introduction risk as a function of parameters that 1) could be predicted within our shipping and environmental dataset in a global scale and 2) could be estimated with empirical data. Other parameters that are known to influence biofouling introduction risk which could not be included in this study include:

- We do not take voyage conditions into consideration (i.e., ships traveling through the freshwater canal, cold/icy Arctic)
- We do not try to estimate dry-docking (i.e., many ships in the Great Lakes are taken out of the water to be cleaned during the winter when it is icy).

- We do not include current biofouling policies (i.e. New Zealand).
- The limited empirical data we do have is focused on macro-invertebrates.

2 Supplementary Results

2.1 Higher-order nodes in ballast SF-HON

As mentioned in the manuscript (section 2.1 and 2.3), ballast water creates significantly more higher-order nodes in SF-HON, compared to biofouling. The degree distribution (the probability distribution of node degrees in the entire network) [19] of the ballast SF-HON also changes after extracting the higher-order dependencies, while the degree distribution of biofouling SF-HON does not alter significantly between the SF-FON and SF-HON (Figure S3 (a) and Figure S3 (b), note that the y-axis is in log scale). There are significantly more nodes of small degree in ballast SF-HON compared to the ballast SF-FON, while the number of such nodes is similar among the biofouling SF-HON and biofouling SF-FON. The additional nodes in the ballast SF-HON are higher-order nodes that result in higher-order introduction pathways.

Furthermore, by comparing Figure S3 (c) and Figure S3 (d) we notice that the average clustering spectrum (average clustering coefficient (CC) of all nodes with degree k where CC is a measure of the overall level of clustering in a network [20]) of ballast SF-HON is smaller than that of the SF-FON version, indicating the sparsity of the ballast SF-HON caused by a significantly larger number of nodes.

Significant higher-order patterns in ballast SF-HON motivated us to analyze the distribution of higher-order nodes across separate realms. Figure S5 shows this distribution for 8 major realms (we show the results up to fifth-order for simplicity, but dependency orders up to 15_{th} exists in the network). We observe that the Arctic contains the highest percentage of first-order nodes (84.07% of the Arctic nodes). We notice that Western Indo-Pacific, Central Indo-Pacific, and Temperate Northern Atlantic contain a higher percentage of higher-order nodes. Temperate Northern Atlantic contains the largest portion (50.49%) of all the nodes in ballast SF-HON, and 84.62% of the nodes in this realm are of second order or higher. After that, Central Indo-Pacific contains 15.55% of all the nodes, of which 77.32 % are of second order or higher.

2.2 Evolution of SF-HONs and SF-FONs

As discussed in section 3.3, number of nodes and edges changes differently in Sf-HONs and SF-FONs. In this section we analyze the variations in network density for all four models. Figure S4 shows that ballast and biofouling SF-FONs show a similar trend in network density over time, while SF-HONs show different patterns. In particular, ballast SF-HON became denser in 2008, while biofouling SF-HON became less dense in this year. However, the increase in the density of ballast SF-HON is not due to increase in the number of edges, but due to significant decrease in the number of nodes (as seen in Figure 6) and as a result, a decrease in the number of higher-order patterns as discussed in section 3.3. On the other hand, in biofouling SF-HON, the number of higher-order nodes have increased and number of edges decreases, resulting in a decrease in the network density in 2008.

2.3 Evaluation of SF-HON and SF-FON

In this section, we provide a detailed breakdown of NIS spread risk error for each SF-HON. Figure S8 displays the breakdown of error using the six network models, for each country and state. We can see that across all datasets, SF-HONs perform generally better than SF-FONs and All-Paths models. Figure S8 (c) displays the result for the AquaNIS dataset. In this data, biofouling SF-HON has the lowest error. Figure S8 (b) corresponds to the NEMESIS data for United States. Figure S8 (a) corresponds to USGS

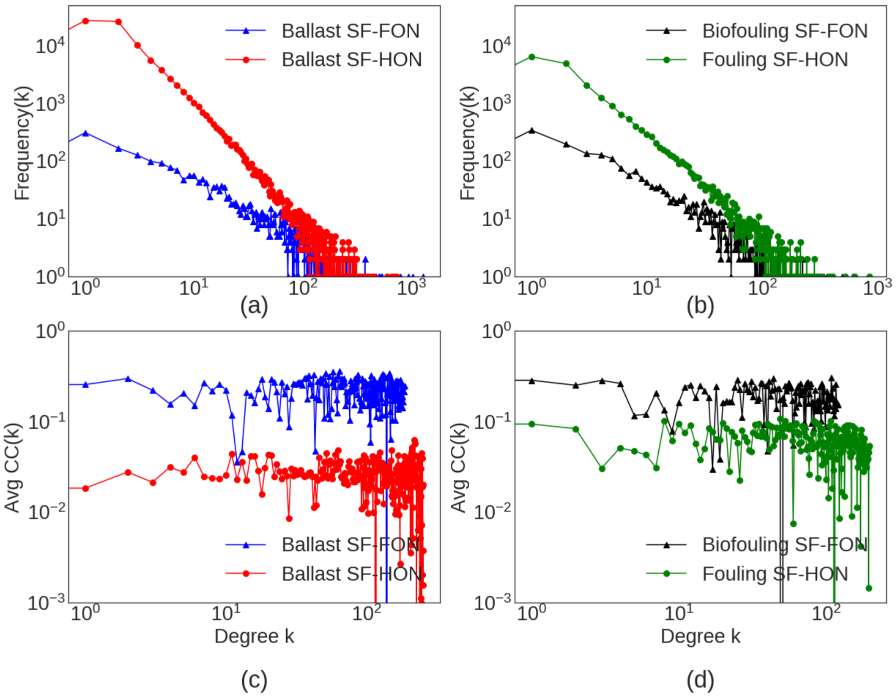

Figure S3: Comparison of degree distribution and clustering spectrum of the SF-HON and SF-FON version of the two risk networks. There are significantly more higher-order nodes, resulting in a much sparser network compared to the first-order version (a). The average CC for ballast SF-HON is significantly lower than the first-order version (c). Such patterns are not as significant in biofouling SF-HON, as observed in (b) and (d).

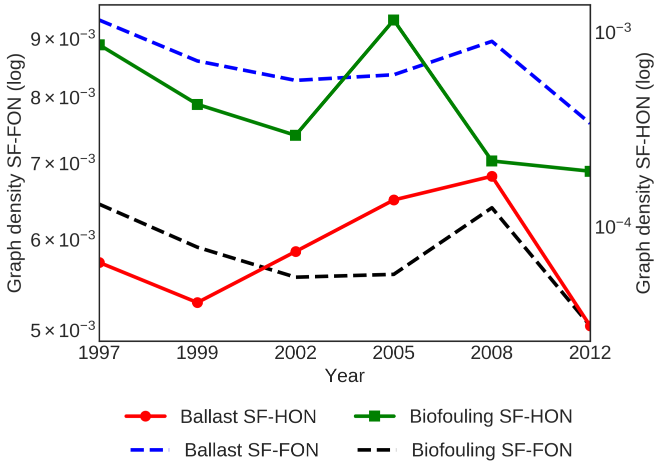

Figure S4: Variation of graph density for all network models. SF-FONs show a similar trend in network density, while SF-HONs show different patterns for network density over the years. Note that for SF-FONs and SF-HONs different axis are used.

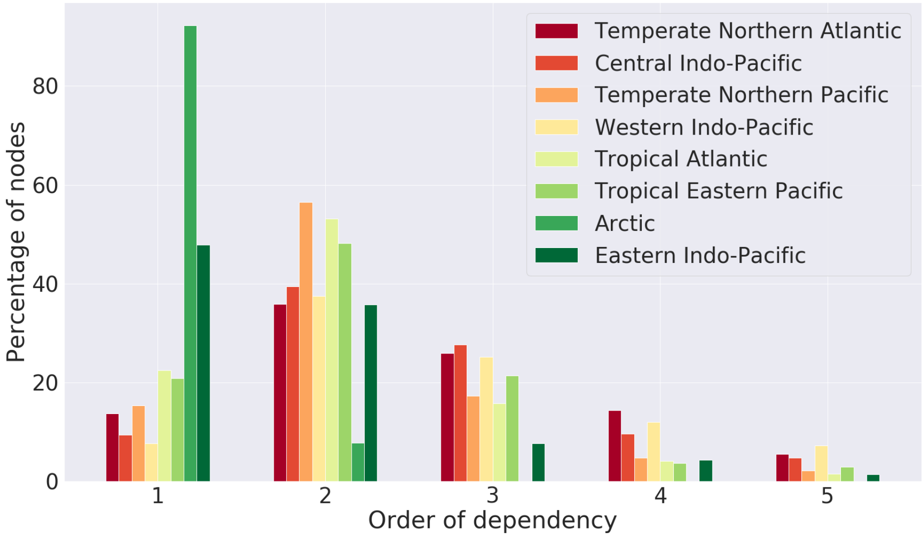

Figure S5: Variation of dependency orders in ballast risk network across major realms. Orders higher than five are not shown for demonstration purpose. The realms are ordered based on the median order of dependency.

Ballast risk network		Biofouling risk network	
Port	# of clusters	Port	# of clusters
Singapore (Singapore)	191	Ulsan (South Korea)	80
Gibraltar (UK)	173	Singapore (Singapore)	52
Tarifa (Spain)	98	San Antonio (Chile)	26
Cape Finisterre (Spain)	83	Balikpapan (India)	20
Santa Marta (Colombia)	82	Jakarta (India)	19
Richards Bay (South Africa)	79	Penang (Malaysia)	16
Haugesund (Norway)	79	Map Ta Phut (Thailand)	15
Halifax (Canada)	64	Ko Sichang (Thailand)	15
Istanbul (Turkey)	64	Visakhapatnam (India)	14
Port Said (Egypt)	63	Fujairah (United Arab Emirates)	14
Vancouver (Canda)	60	Halifax (Canada)	14
Hong Kong (china)	59	Conakry (Guinea)	13

Table S1: Ports with the highest NIS spread risk along with the number of clusters they belong to. 62% of the high-risk ports in ballast SF-HON belong to Central-Indo Pacific realm. While in biofouling SF-HON, this number is 25%, and 56% of the high-risk ports belong to the Temperate Northern Atlantic realm.

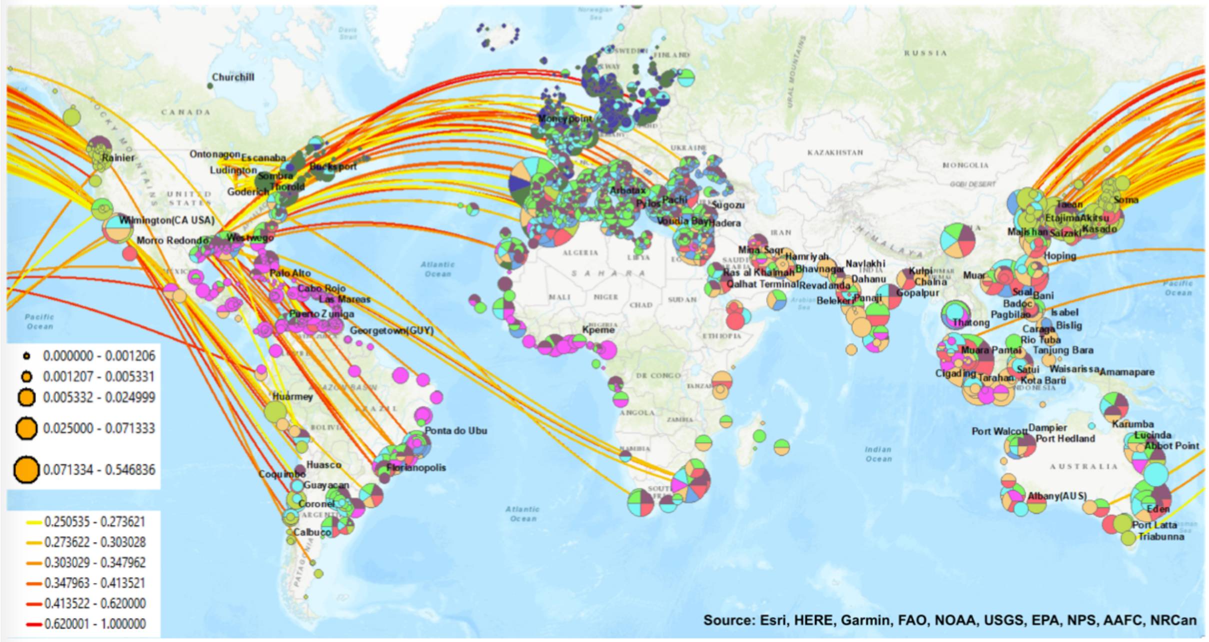

(a)

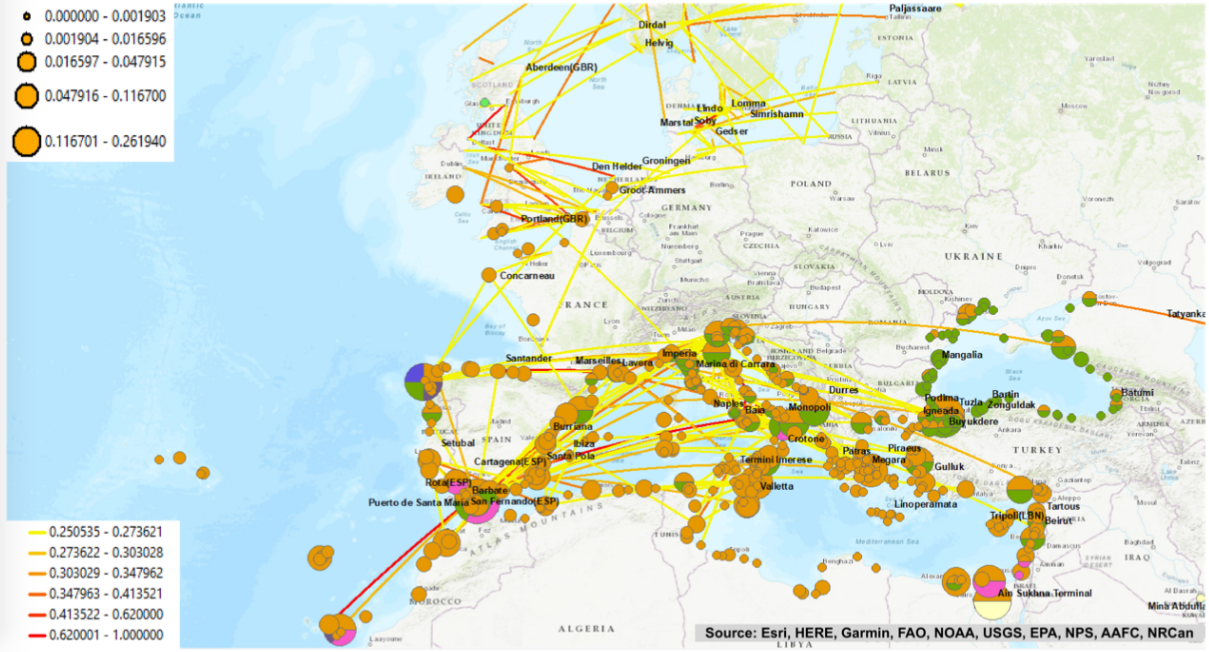

(b)

Figure S6: Main introduction pathways of North America through ballast discharge (a) and main introduction pathways of Europe through biofouling (b). High risk connections are displayed darker, while low-risk connections are displayed lighter. The coloring of the ports indicates the port cluster. Ports which belong to multiple clusters through the higher-order patterns are shown as pie chart with multiple colors. The pie chart size indicates the relative NIS spread risk for the port.

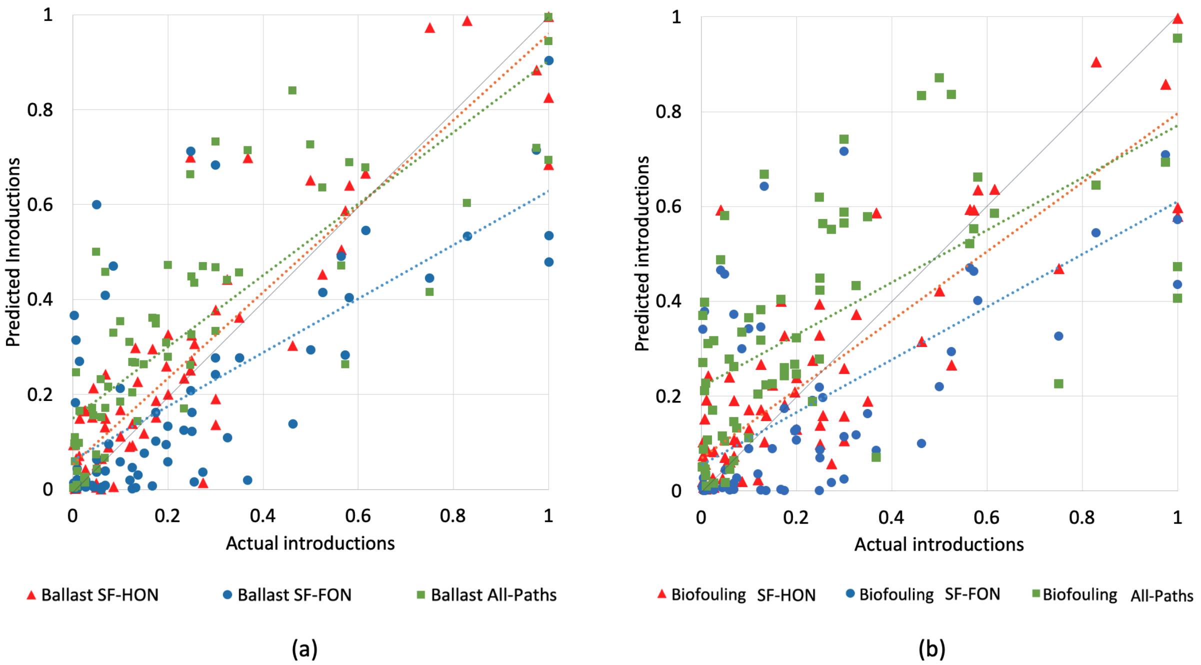

Figure S7: Performance of each network model for ballast (a) and biofouling introductions (b). The x-axis shows the actual introductions and the y-axis shows the predicted values. The overall trend for each model is shown with a linear line and the grey straight line shows the performance of an ideal model. SF-FONs tend to under-estimate the risk while All-Paths models tend to over-estimate the risk.

Compared models	NAS	NEMESIS	AquaNIS
Biofouling (SF-HON , SF-FON)	0.147	**0.0453**	**0.010**
Biofouling (SF-HON , All-Paths)	**0.036**	**0.0073**	**0.035**
Ballast (SF-HON , SF-FON)	**0.020**	0.1048	0.101
Ballast (SF-HON , All-Path)	0.406	**0.0466**	0.295

Table S2: p-values obtained from the t-tests performed between SF-HON models and the two other models.

dataset in which ballast SF-HON has a lower error across all states. We further illustrate the prediction of each model versus the actual introductions in Figure S7. Overall, we observe an under-estimation of the risk by SF-HON predictions and over-estimation of the risk by All-Paths model predictions. It is important to note that, some states/countries had fewer records resulting in higher prediction error and higher variance, which is a limitation of our work.

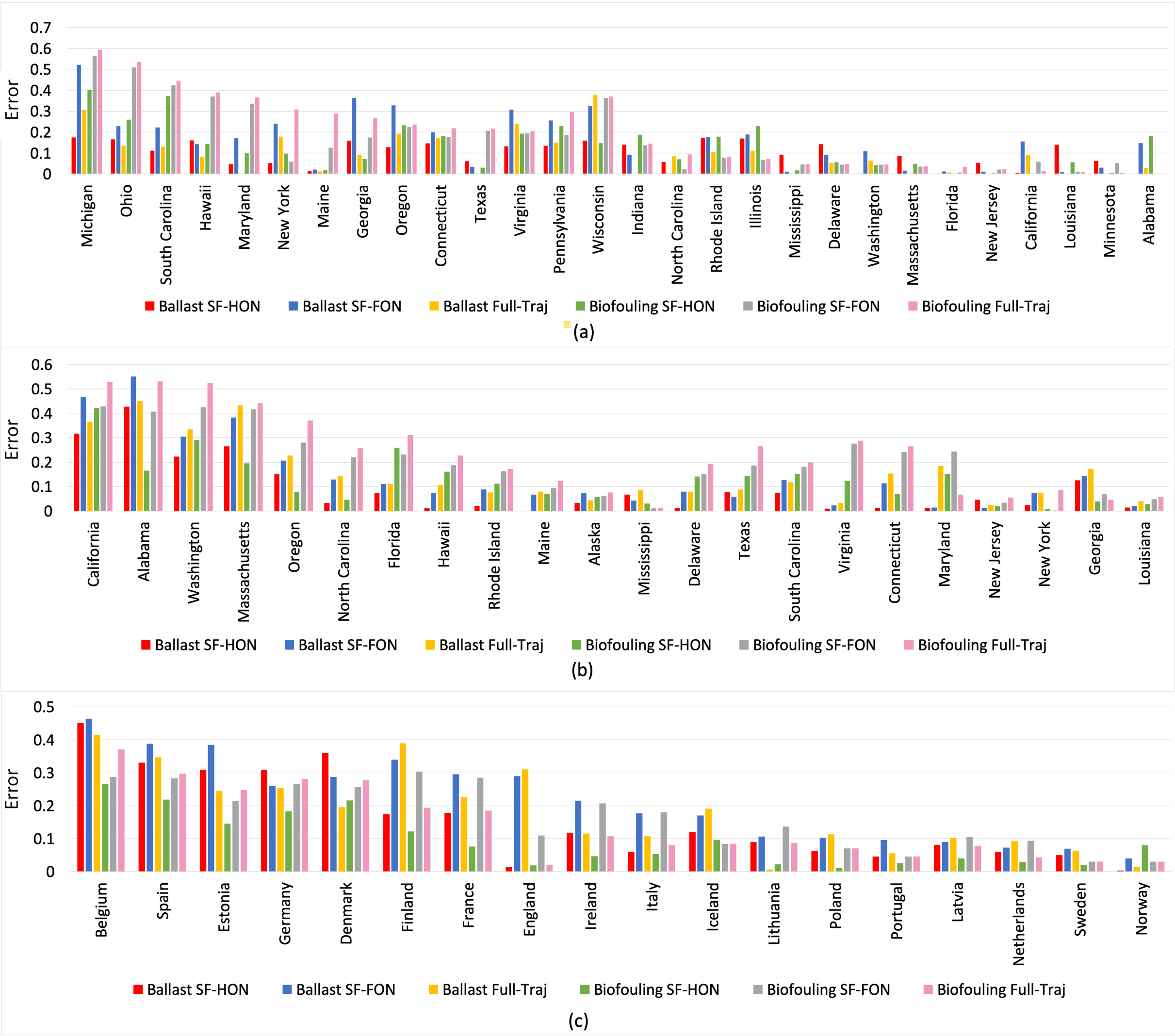

Figure S8: Breakdown of evaluation results for each state and country. (a) USGS dataset for the US, (b) NEMESIS dataset for US, (c): AquaNIs dataset for Europe.

References

[1] Drake JM, Lodge DM. Global hot spots of biological invasions: evaluating options for ballast–water management. Proceedings of the Royal Society of London B: Biological Sciences. 2004;271(1539):575–580.

[2] Kaluza P, Kölzsch A, Gastner MT, Blasius B. The complex network of global cargo ship movements. Journal of the Royal Society Interface. 2010;7(48):1093–1103.

[3] Kölzsch A, Blasius B. Indications of marine bioinvasion from network theory. The European Physical Journal B. 2011;84(4):601–612.

[4] Seebens H, Gastner M, Blasius B. The risk of marine bioinvasion caused by global shipping. Ecology letters. 2013;16(6):782–790.

[5] Xu J, Wickramarathne TL, Chawla NV, Grey EK, Steinhaeuser K, Keller RP, et al. Improving management of aquatic invasions by integrating shipping network, ecological, and environmental data: data mining for social good. In: Proceedings of the 20th ACM SIGKDD international conference on Knowledge discovery and data mining. ACM; 2014. p. 1699–1708.

[6] Muirhead JR, Minton MS, Miller WA, Ruiz GM. Projected effects of the Panama Canal expansion on shipping traffic and biological invasions. Diversity and Distributions. 2015;21(1):75–87.

[7] Eldredge LG, Carlton JT. Hawaiian marine bioinvasions: a preliminary assessment. Pacific Science. 2002;56(2):211–212.

[8] Kospartov M, Inglis G, Seaward K, van den Brink A, DArchino R, Ahyong S. Non-indigenous and cryptogenic marine species in New Zealand–Current state of knowledge: Interim report. Report prepared for MAFBNZ project BNZ10740 National Institute of Water and Atmospheric Research, Wellington. 2008;.

[9] Hewitt CL, Campbell ML, Thresher RE, Martin RB, Boyd S, Cohen BF, et al. Introduced and cryptogenic species in Port Phillip Bay, Victoria, Australia. Marine Biology. 2004;144(1):183–202.

[10] Gollasch S. The importance of ship hull fouling as a vector of species introductions into the North Sea. Biofouling. 2002;18(2):105–121.

[11] Fofonoff PW, Ruiz GM, Steves B, Carlton JT. In ships or on ships? Mechanisms of transfer and invasion for nonnative species to the coasts of North America. Invasive species: vectors and management strategies. 2003;152:162–169.

[12] Schimanski KB, Piola RF, Goldstien SJ, Floerl O, Grandison C, Atalah J, et al. Factors influencing the en route survivorship and post-voyage growth of a common ship biofouling organism, Bugula neritina. Biofouling. 2016;32(8):969–978.

[13] Canning-Clode J, Fofonoff P, Riedel GF, Torchin M, Ruiz GM. The effects of copper pollution on fouling assemblage diversity: a tropical-temperate comparison. PloS one. 2011;6(3):e18026.

[14] Freestone AL, Osman RW. Latitudinal variation in local interactions and regional enrichment shape patterns of marine community diversity. Ecology. 2011;92(1):208–217.

[15] Schoener A, Long ER, DePalma J. Geographic variation in artificial island colonization curves. Ecology. 1978;59(2):367–382.

[16] Canning-Clode J, Maloney KO, McMahon SM, Wahl M. Expanded view of the local–regional richness relationship by incorporating functional richness and time: a large-scale perspective. Global Ecology and Biogeography. 2010;19(6):875–885.

[17] Scianni C, Brown C, Newsom A, Nedelcheva R, Falkner M, Dobroski N. 2013 Biennial Report on the California Marine Invasive Species Program; 2013.

[18] Coutts AD, Piola RF, Taylor MD, Hewitt CL, Gardner JP. The effect of vessel speed on the survivorship of biofouling organisms at different hull locations. Biofouling. 2010;26(5):539–553.

[19] Barabási AL. A.-L. Barabási and R. Albert, Science 286, 509 (1999). Science. 1999;286:509.

[20] Watts DJ, Strogatz SH. Collective dynamics of small-worldnetworks. nature. 1998;393(6684):440.
